# Thermal Rearrangement of Thiocarbonyl-Stabilised Triphenylphosphonium Ylides Leading to (*Z*)-1-Diphenylphosphino-2-(phenylsulfenyl)alkenes and Their Coordination Chemistry

**DOI:** 10.3390/molecules29010221

**Published:** 2023-12-31

**Authors:** R. Alan Aitken, Graham Dawson, Neil S. Keddie, Helmut Kraus, Heather L. Milton, Alexandra M. Z. Slawin, Joanne Wheatley, J. Derek Woollins

**Affiliations:** EaStCHEM School of Chemistry, University of St Andrews, North Haugh, St. Andrews, Fife KY16 9ST, UK

**Keywords:** flash vacuum pyrolysis, phosphonium ylide, phosphine, hemilabile ligand, transition metal complex, X-ray structure

## Abstract

While thiocarbonyl-stabilised phosphonium ylides generally react upon flash vacuum pyrolysis by the extrusion of Ph_3_PS to give alkynes in an analogous way to their carbonyl-stabilised analogues, two examples with a hydrogen atom on the ylidic carbon are found to undergo a quite different process. The net transfer of a phenyl group from P to S gives (*Z*)-configured 1-diphenylphosphino-2-(phenylsulfenyl)alkenes in a novel isomerisation process via intermediate λ^5^-1,2-thiaphosphetes. These prove to be versatile hemilabile ligands with a total of seven complexes prepared involving five different transition metals. Four of these are characterised by X-ray diffraction with two involving the bidentate ligand forming a five-membered ring metallacycle and two with the ligand coordinating to the metal only through phosphorus.

## 1. Introduction

**Scheme 1:**
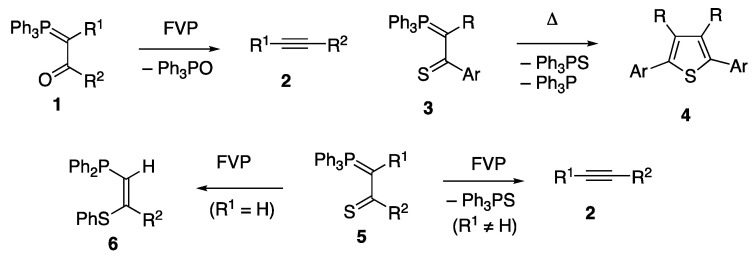
Previously reported thermal reactivity of carbonyl- and thiocarbonyl-stabilised phosphonium ylides.

The thermal extrusion of Ph_3_PO from carbonyl-stabilised triphenylphosphonium ylides **1** is a well-established synthetic route to functionalised alkynes **2** (Scheme 1) [1,2,3]. The process proceeds particularly well using flash vacuum pyrolysis (FVP), and we have found that the phosphorus to carbonyl coupling constant ^2^*J*_P–CO_ provides a diagnostic parameter for the likely success of the reaction, with ylides for which ^2^*J*_P–CO_ < 11 Hz usually providing the alkynes in high yield [4]. By way of contrast, the thermal behaviour of the corresponding thiocarbonyl-stabilised ylides **3** has only been examined in a few cases and Bestmann and Schaper found that heating the ylides **3** above their melting point resulted in a bimolecular process with the loss of Ph_3_P and Ph_3_PS to give tetrasubstituted thiophenes **4** [5]. Some time ago, we described a preliminary study in which FVP of the ylides **5** was found to proceed as expected by analogy with the carbonyl analogues **1** with the loss of Ph_3_PS to give alkynes **2** for R^1^ ≠ H, but when R^1^ was hydrogen, a quite different process was observed: rearrangement with the transfer of a phenyl group from P to S giving the potentially useful bidentate proligands **6** [6]. In this paper, we describe in more detail the synthesis and structure of these novel phosphinovinyl sulfides as well as their coordination chemistry.

## 2. Results and Discussion

**Figure 1:**
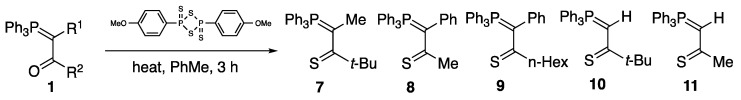
Thiocarbonyl ylides used in this study.

Synthetic access to thiocarbonyl-stabilised ylides **5** is available using various methods including the treatment of non-stabilised ylides with dithioesters or dithiocarbonates [7], or activation of the corresponding carbonyl-stabilised ylides with triflic anhydride [8] or POCl_3_ [9] followed by treatment with sodium sulfide. For the current study, we used the direct reaction of carbonyl-stabilised ylides **1** with Lawesson’s reagent introduced by Capuano and coworkers [10], and in this way, we prepared the five examples **7**–**11** (Figure 1) from their carbonyl analogues.

Compounds **7**–**9** are already known [8] andgave analytical and spectroscopic data in agreement with the reported values. The new compounds **10** and **11** were fully characterised and showed distinctive NMR signals confirming the presence of P=CH–C=S [**10** δ_P_ +5.0; δ_H_ 5.22 (d, ^2^*J* 34 Hz, P=CH); δ_C_ 81.3 (d, ^1^*J* 118 Hz, P=CH), 214.4 (d, ^2^*J* 4 Hz, C=S). **11** δ_P_ +8.1; δ_H_ 5.18 (d, ^2^*J* 32 Hz, P=CH); δ_C_ 84.1 (d, ^1^*J* 113 Hz, P=CH), 200.5 (d, ^2^*J* 4 Hz, C=S)]. In fact, the phosphorus coupling extended throughout the structures with all carbon signals except the CH_3_ of *t*-butyl observed as doublets in the ^13^C NMR spectra. This is discussed further below in the context of a comparison of the coupling pattern before and after the thermal rearrangement. The structure of **10** was also confirmed by X-ray diffraction (CSD RefCode: AJOMUI) and this was described in our earlier communication [6]. We might also note at this point that compound **10** has a particularly pungent and unpleasant smell. Although it is not very volatile, it is extremely persistent and inadvertent contact with equipment or work surfaces contaminated with **10**, even after several months, results in the release of its characteristic smell. We speculate that this may be due to slow hydrolysis of the P=C bond to give Ph_3_PO and release thiopinacolone, *t*-BuC(=S)Me.

When the thiocarbonyl ylides **7**, **8** and **9** were subjected to FVP, there was complete reaction at a furnace temperature of 650 °C to give Ph_3_PS (δ_P_ +43) at the furnace exit and the expected alkynes **2**, 2,2-dimethylpent-3-yne, 1-phenylpropyne and 1-phenyloctyne, in the cold trap. Thus, these compounds behave in a similar way to their carbonyl analogues but react more readily than the latter, which require a temperature of 750 °C for complete reaction [11]. The higher reactivity of thiocarbonyl- as compared to carbonyl-stabilised ylides is a feature that we have already noted and quantified in a series of kinetic studies on the pyrolysis of carbamoyl and thiocarbamoyl ylides [12,13,14].

**Scheme 2:**
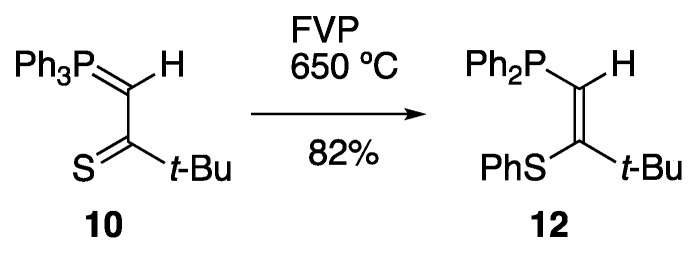
FVP of **10** to give **12**.

When the two ylides **10** and **11** were subjected to FVP, the reaction was also complete at 650 °C, but the process involved turned out to be completely different. In each case, only a single main product was obtained, which was isomeric with the starting material. In the case of **10**, the product **12** was obtained in good yield and in a pure form as a crystalline solid after preparative TLC. This showed a ^31^P NMR signal at −19.8 ppm, in the region expected for an alkyldiphenylphosphine, and the single P–CH= hydrogen gave a ^1^H NMR singlet at 6.94 ppm (Scheme 2).

**Figure 2:**
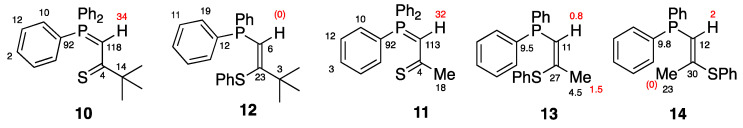
Magnitude of P–H (red) and P–C (black) coupling constants (Hz).

The ^13^C NMR spectrum, and particularly the pattern of P–C coupling, was particularly informative and showed major changes from the values in **10** (Figure 2).

The values of *J*_P–C_ for the thiocarbonyl ylides **10** and **11** are consistent with those well established for the corresponding carbonyl ylides [11] and carbamoyl/thiocarbamoyl ylides [12,13,14]. However, in the rearranged product **12**, the values around P-Ph are more similar to those in Ph_3_P, and the much higher coupling to P–C=C as compared to P–C=C as well as the absence of coupling to P–CH are surprising features. As already mentioned in our preliminary communication [6], the structure of **12** was confirmed by X-ray diffraction (CSD RefCode: AJOMOC).

**Scheme 3:**
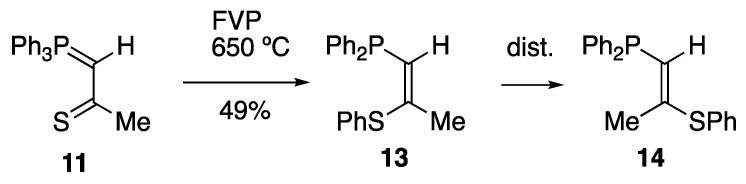
FVP of **11** to give **13** and **14**.

When we examined the corresponding pyrolysis of the thioacetyl ylide **11**, the corresponding reaction occurred at the same temperature, but the product was now a liquid formed in lower yield and containing some impurities including Ph_3_P, Ph_3_PO and Ph_3_PS. More interestingly, while it was predominantly (6.5:1) the (*Z*)-isomer **13** (δ_P_ −22.7), signals attributed to the (*E*)-isomer **14** (δ_P_ −25.4) were also apparent (Scheme 3).

**Figure 3:**
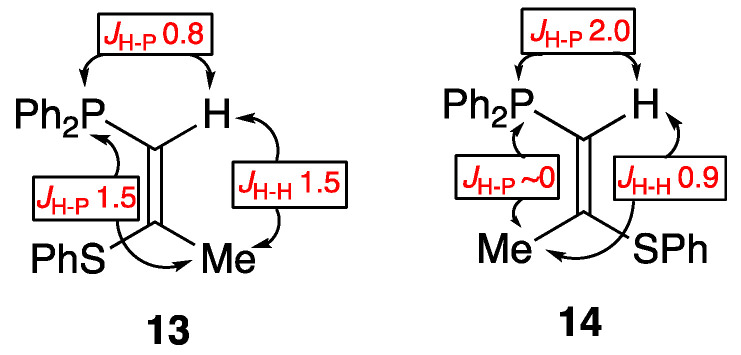
Coupling constants in ^1^H NMR spectra of **13** and **14**.

Attempts to purify this by repeated Kugelrohr distillation under reduced pressure led instead to isomerisation to give a 1:1 mixture of **13** and **14**. At first sight, it might seem surprising that the product is obtained mainly as the less thermodynamically stable isomer from pyrolysis at 650 °C but then isomerises to the more stable isomer upon simple distillation at 90 °C, but this only serves to emphasise the mild nature of the flash vacuum pyrolysis technique. In addition, it has been shown that under FVP conditions, (*Z*)-alkenes do not normally isomerise to the (*E*)-isomer to any great extent at temperatures as low as 650 °C with the degree of conversion of (*Z*)- to (*E*)-stilbene, for example, being determined as 12%, in good agreement with our results [15]. The presence of aromatic impurities even after distillation prevented full assignment of the ^13^C NMR spectra for **13** and **14**, but the key signals and the values of the phosphorus coupling constants (Figure 2) were in good agreement with those for **12** while also showing significant differences between the two isomers. The detailed form of the signals for P–CH and P–C=C–Me in the two isomers was at first sight surprising. However, this could be explained by coincidental equivalence of some H–H and P–H coupling constants, and the observed patterns (see Supplementary Materials, Figure S14) could be satisfactorily simulated using the values shown in Figure 3.

**Figure 4:**
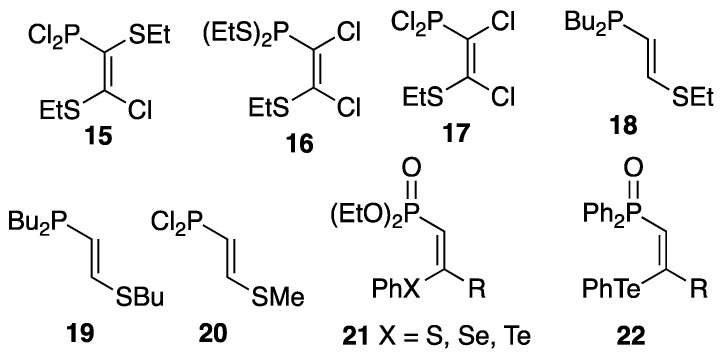
Some previously reported P–C=C–S proligands.

Although the 1,2-arrangement of phosphine and sulfide functions on an alkene double bond, particularly in the (*Z*)-configuration, gives potentially valuable “hemilabile” proligands, few such compounds seem to be known (Figure 4). Chlorinated compounds such as **15** [16], **16** [17] and **17** [18] have been prepared as mixtures of (*E*)- and (*Z*)-isomers by the addition of phosphorus compounds to alkynes. The simpler disubstituted alkenes **18** [19], **19** [20] and **20** [21] have also been prepared but these are the (*E*)-isomers as shown. More recently, the (*Z*)-configured vinylphosphonates **21** containing sulfide, selenide and telluride functions [22] as well as the tellurovinylphosphine oxides **22** [23] have also been reported.

**Scheme 4:**
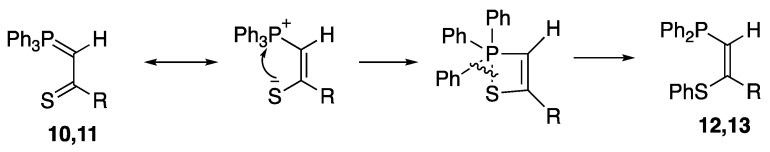
Mechanism proposed for the thermal rearrangement of **10** and **11**.

In terms of the mechanism of this new thermal rearrangement, we envisage attack of the nucleophilic sulfur at phosphorus to give a transient λ^5^-1,2-thiaphosphete, which is of course the same intermediate involved in the extrusion of Ph_3_PS to give alkynes as observed for **7**–**9**. This behaviour is also consistent with that of the isolable thiaphosphete **23,** which fragments with the loss of an alkyne to give the benzoxaphosphole *P*-sulfide [24]. However, perhaps due to the relief of steric congestion, the thiaphosphetes derived from **10** and **11** instead undergo what is effectively a reductive elimination at phosphorus to give the (*Z*)-phosphinovinyl sulfides **12** and **13** (Scheme 4).

**Scheme 5:**
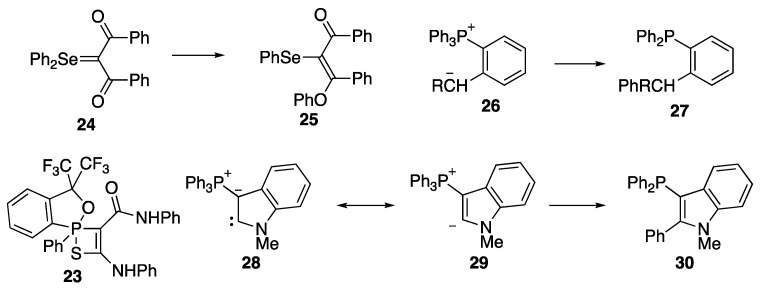
Some mechanistic precedents.

Somewhat similar processes are the transfer of Ph from Se to O in acylselenonium ylides such as **24** to give **25** observed by Rakitin [25] (Scheme 5), the transformation of **26** into **27** postulated by Zbiral in the interaction of ylides Ph_3_P=CHR with benzyne [26] and the rearrangement of the ylide-containing N-heterocyclic carbene **28** via **29** to give 3-phosphinoindole **30** [27].

**Scheme 6:**
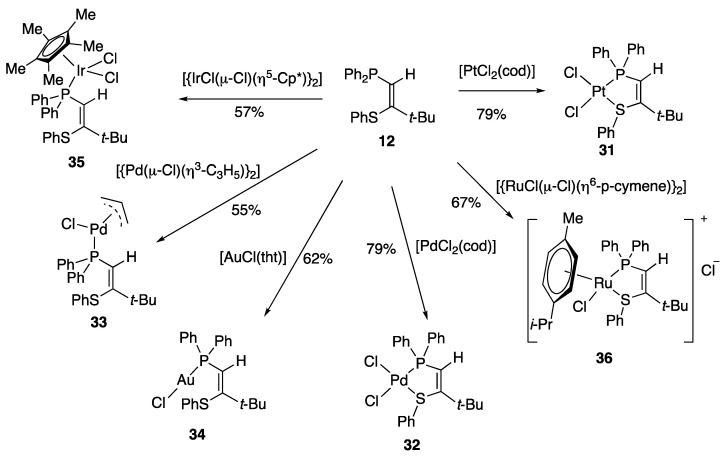
Formation of transition metal complexes from **12**.

With the two new hemilabile proligands in hand, we now set about exploring their coordination chemistry. In our preliminary communication [6], the formation of the square planar platinum complex **31** from **12** was described along with its X-ray structure determination (CSD RefCode: AJONAP). We were also successful in obtaining complexes of **12** with a wide range of other standard transition metal reagents (Scheme 6).

Reaction of the starting materials in CH_2_Cl_2_ followed by partial evaporation and precipitation with diethyl ether gave the new complexes **32**–**36** in moderate to good yield as crystalline solids, giving the expected microanalytical data and ^1^H and ^31^P NMR spectra. From the analytical data, it was clear that the complexes had formed with the expected stoichiometry according to the metal source employed, with **12** acting as a bidentate ligand in the square planar platinum(II) and palladium(II) complexes **31** and **32** and the cationic ruthenium(II) complex **36**, but as a monodentate ligand through the more strongly donating phosphorus atom in palladium(II) complex **33**, gold(I) complex **34** and iridium(III) complex **35**. In addition to the compound **31** already confirmed by X-ray diffraction [6], we were able to obtain X-ray structures for gold complex **34** and iridium complex **35** (see below).

**Scheme 7:**
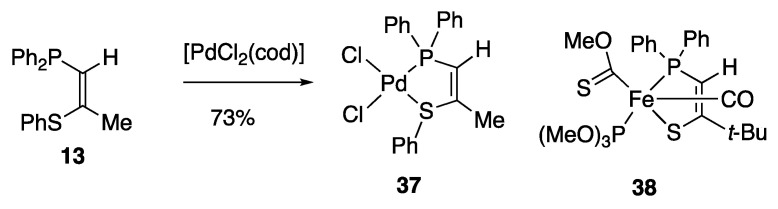
Formation of palladium complex **37** and structure of a related iron complex.

Although the methyl proligand **13** was available in lower quantity and purity, we were able to prepare its palladium dichloride complex **37** analogous to **32** and also determined its X-ray structure (Scheme 7).

**Figure 5:**
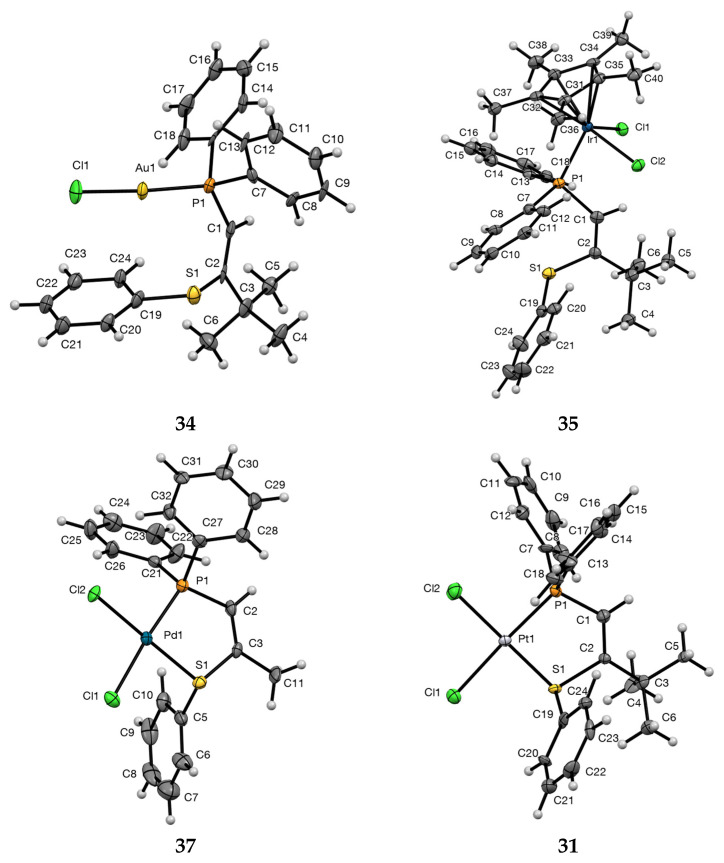
X-ray structures of complexes **34**, **35**, **37** and **31** showing probability ellipsoids at 50% level and numbering systems used.

The structures of the complexes **34**, **35** and **37** together with the numbering systems used are shown below (Figure 5) with that of **31** [6] also included for comparison.

**Figure 6:**
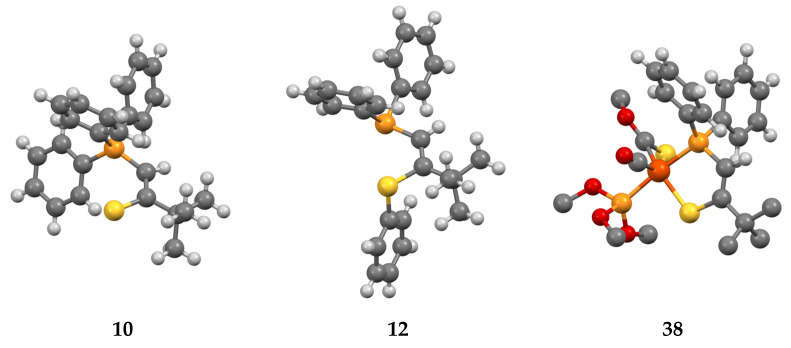
Published structures of **10**, **12** and **38.**

For comparison, we also show (Figure 6) the previously reported structures of **10** [6] and **12** [6], and the iron(II) complex **38** [28], which, although made in a quite different way, contains the phosphinovinylthiolate corresponding to **12** as an anionic bidentate ligand.

The key structural parameters for complexes **34**, **35** and **37** and, for comparison, those for **10**, **12**, **31** and **38** are presented in Table 1. In all seven structures, the P–C–C–S fragment is quite accurately planar with a torsion angle of <10° in every case.

If we first compare **10** and **12**, the major change in geometry associated with the transformation of P=C–C=S into P–C=C–S is clear. However, comparing the structural parameters of **12** with those of its complexes **31**, **34** and **35** as well as the related thiolate complex **38** shows a remarkable degree of consistency. As expected, the complexes **34** and **35** where the metal binds only to phosphorus have bond lengths around the =C–SPh that are relatively unaffected, while the bidentate binding to platinum in **31** results in the significant lengthening of C–S and shortening of C–P. While the smaller size of AuCl means the ligand **12** can retain the orientation of the PPh_2_ group, coordination to the much larger Cp*IrCl_2_ requires the PPh_2_ group to rotate, placing the phenyl groups facing towards SPh. The similarity between the geometry of the neutral ligand **12** in complexes such as **31** and the anionic enethiolate in **38** is also notable, with only the =C–S length being significantly shorter in the latter. The angles within the five-membered ring in complexes **31**, **37** and **38** are also remarkably consistent.

## 3. Experimental Section

### 3.1. General Experimental Details

NMR spectra were recorded on solutions in CDCl_3_ unless otherwise stated using Bruker instruments, and chemical shifts are given in ppm to high frequency from Me_4_Si for ^1^H and ^13^C and H_3_PO_4_ for ^31^P with coupling constants *J* in Hz. The ^13^C NMR spectra are referenced to the solvent signal at 77.0 (CDCl_3_). IR spectra were recorded on a Perkin Elmer 1420 instrument. Elemental analysis was conducted using a Carlo Erba CHNS analyser. Mass spectra were obtained using a Micromass instrument and the ionisation method used is noted in each case. Preparative TLC was carried out using 1.0 mm layers of Merck alumina 60 G containing 0.5% Woelm fluorescent green indicator on glass plates. Melting points were recorded on a Gallenkamp 50W melting point apparatus or a Reichert hot-stage microscope.

Flash vacuum pyrolysis (FVP) was carried out in a conventional flow system by subliming the starting material through a horizontal quartz tube (30 × 2.5 cm) externally heated by a tube furnace to 650 °C and maintained at a pressure of 2–5 × 10^−2^ Torr by a rotary vacuum pump. Products were collected in a liquid N_2_ cooled U-shaped trap and purified as noted.

General organic and inorganic reagents and solvents were obtained from standard suppliers and used as received. Dry THF was prepared by storage over sodium wire. Starting transition metal complexes [AuCl(tetrahydrothiophene)] [29], [PdCl_2_(cyclooctadiene)] [30], [PtCl_2_(cyclooctadiene)] [31], [{RuCl(μ-Cl)(η^6^-*p*-MeC_6_H_4_^i^Pr)}_2_] [32], [{IrCl(μ-Cl)(η^5^-C_5_Me_5_)}_2_] [33] and [{Pd(m-Cl)(η^3^-C_3_H_5_)}_2_] [34] were prepared by the reported methods.

### 3.2. Preparation of Thiocarbonyl Ylides

#### 3.2.1. Preparation of Thiopivaloylmethylenetriphenylphosphorane **10**

A solution of pivaloylmethylenetriphenylphosphorane (5.0 g, 13.9 mmol) and Lawesson’s reagent (2.81 g, 6.9 mmol) in toluene (300 mL) was heated under reflux under nitrogen for 3 h. The mixture was allowed to cool to RT and the solution was poured off leaving an insoluble oily residue and evaporated. Recrystallisation of the resulting solid from ethyl acetate gave the product (2.86 g, 55%) as pale-yellow crystals, mp 200–202 °C; (Found: C, 76.5; H, 6.4. C_24_H_25_PS requires C, 76.6; H, 6.7%); ν_max_/cm^−1^ 1572, 1260, 1205, 1160, 1105, 978, 880, 792, 751, 722, 713, 691 and 620; ^1^H NMR (300 MHz) δ_H_ 1.40 (9H, s), 5.22 (1H, d, *J* 34, CH=P), 7.40–7.48 (6H, m), 7.48–7.52 (3H, m) and 7.67–7.80 (6H, m); ^13^C NMR (75 MHz) δ_C_ 31.3 (3C), 43.2 (d, *J* 14, *C*Me_3_), 81.3 (d, *J* 118, P=C), 125.3 (d, *J* 92, C-1 of Ph), 128.5 (d, *J* 12, C-3 of Ph), 131.6 (d, *J* 2, C-4 of Ph), 132.8 (d, *J* 10, C-2 of Ph) and 214.4 (d, *J* 4, C=S); ^31^P NMR (121 MHz) δ_P_ +5.0; MS (EI) *m/z* 376 (M^+^, 16%), 343 (9), 319 (100), 294 (7), 262 (12) and 183 (23).

#### 3.2.2. Preparation of Thioacetylmethylenetriphenylphosphorane **11**

A solution of acetylmethylenetriphenylphosphorane (8.0 g, 25 mmol) and Lawesson’s reagent (5.1 g,12.6 mmol) in toluene (300 mL) was heated under reflux under nitrogen for 3 h. The mixture was allowed to cool to RT and the solution was poured off leaving an insoluble oily residue and evaporated. Recrystallisation of the resulting solid from ethyl acetate gave the product (4.62 g, 55%) as pale-yellow crystals, mp 172–174 °C; (Found: C, 75.2; H, 5.3. C_21_H_19_PS requires C, 75.4; H, 5.7%); ν_max_/cm^−1^ 1585, 1270, 1175, 1106, 993, 872, 763, 747, 723, 686 and 660; ^1^H NMR (300 MHz) δ_H_ 2.63 (3H, s), 5.18 (1H, d, *J* 32, CH=P), 7.45–7.55 (6H, m), 7.55–7.65 (3H, m) and 7.70–7.80 (6H, m); ^13^C NMR (75 MHz) δ_C_ 36.8 (d, *J* 18, Me), 84.1 (d, *J* 113, P=C), 124.6 (d, *J* 92, C-1 of Ph), 128.9 (d, *J* 12, C-3 of Ph), 132.3 (d, *J* 3, C-4 of Ph), 133.3 (d, *J* 10, C-2 of Ph) and 200.5 (d, *J* 4, C=S); ^31^P NMR (121 MHz) δ_P_ +8.1; MS (EI) *m/z* 334 (M^+^, 85%), 319 (16), 301 (40), 262 (14), 225 (30), 183 (38) and 43 (100).

### 3.3. Thermal Rearrangement of Thiocarbonyl Ylides

#### 3.3.1. Preparation of (Z)-1-Diphenylphosphino-3,3-dimethyl-2-phenylthiobut-1-ene **12**

FVP of the ylide **10** (0.50 g, 1.33 mmol) was performed at 650 °C and 3.8 × 10^−2^ Torr and was complete within 1 h. Preparative TLC (silica, diethyl ether) of the crude material gave the product **12** (0.41 g, 82%) as pale-yellow plates, mp 121–123 °C; (Found: C, 76.3; H, 6.7; S, 8.2. C_24_H_25_PS requires C, 76.6; H, 6.7; S, 8.5%); ν_max_/cm^−1^ 1582, 1551, 1303, 1181, 1118, 1027, 960, 740, 720 and 694; ^1^H NMR (300 MHz) δ_H_ 1.21 (9H, s), 6.94 (1H, s), 7.05–7.25 (5H, m) and 7.25–7.40 (10H, m); ^13^C NMR (75 MHz) δ_C_ 29.8 (3CH_3_), 41.5 (d, *J* 3, *C*Me_3_), 125.2 (C-4 of SPh), 127.5 (C-3 of SPh), 128.24 (C-4 of PPh), 128.25 (d, *J* 11, C-3 of PPh), 128.6 (C-2 of SPh), 132.6 (d, *J* 19, C-2 of PPh), 137.2 (C-1 of SPh), 137.8 (d, *J* 6, P–CH=), 139.4 (d, *J* 12, C-1 of PPh) and 158.8 (d, *J* 23, S–C=); ^31^P NMR (121 MHz) δ_P_ −19.8; MS (CI) *m/z* 377 (M+H^+^, 100%), 319 (9) and 279 (10).

#### 3.3.2. Preparation of (Z)-1-Diphenylphosphino-2-phenylthiopropene **13**

FVP of the ylide **11** (0.50 g, 78.8 µmol) was performed at 650 °C and 3.8 × 10^−2^ Torr and was complete within 1 h. NMR analysis of the crude product (0.245 g, 49%) showed a 6.5:1 ratio of (*Z*)- and (*E*)-**13**. Repeated Kugelrohr distillation of this (bp 90 °C/0.1 Torr) in an attempt to remove trace impurities of Ph_3_P, Ph_3_PO and Ph_3_PS resulted in isomerisation to afford a 1:1 ratio of (*Z*) and (*E*)-**13**. By comparing the NMR data before and after distillation, the following assignments could be made (owing to peak overlap, definite assignment of the remaining aromatic ^13^C NMR signals was not possible):

(*Z*)-**13**: ^1^H NMR (300 MHz) δ_H_ 2.02 (3H, t, *J* 1.5), 6.33 (1H, qd, *J* 1.5, 0.8) and 7.25–7.75 (15H, m); ^13^C NMR (75 MHz) δ_C_ 148.8 (d, *J* 27, =C–S), 139.0 (d, *J* 9.5, PPh C-1), 132.2 (d, *J* 11, P–CH) and 25.7 (d, *J* 4.5, CH_3_); ^31^P NMR (121 MHz) δ_P_ −22.7.

(*E*)-**13**: ^1^H NMR (300 MHz) δ_H_ 2.23 (3H, d, *J* 0.9), 5.94 (1H, dq, *J* 2.0, 0.9) and 7.25–7.75 (15H, m); ^13^C NMR (75 MHz) δ_C_ 149.8 (d, *J* 30, =C–S), 138.9 (d, *J* 9.8, PPh C-1), 122.0 (d, *J* 12, P–CH) and 20.9 (d, *J* 23, CH_3_); ^31^P NMR (121 MHz) δ_P_ −25.4.

For the isomer mixture (Found: C, 75.2; H, 5.7. C_21_H_19_PS requires C, 75.4; H, 5.7%); ν_max_/cm^−1^ 1584, 1478, 1435, 1184, 1109, 1026, 999, 743, 719 and 694; HRMS (EI) *m*/*z* calcd for C_21_H_19_PS (M^+^) 334.0945. Found 334.0960.

### 3.4. Formation of Transition Metal Complexes

#### 3.4.1. (Z)-1-Diphenylphosphino-3,3-dimethyl-2-phenylthiobut-1-ene Platinum Dichloride Complex **31**

A solution of [PtCl_2_(cod)] (66 mg, 0.18 mmol) in CH_2_Cl_2_ (5 mL) was stirred while a solution of (*Z*)-1-diphenylphosphino-3,3-dimethyl-2-phenylthiobut-1-ene **12** (66 mg, 0.18 mmol) in CH_2_Cl_2_ (5 mL) was added dropwise over 30 min. After 1 h, the mixture was reduced to 1 mL by evaporation, and the addition of diethyl ether (15 mL) led to precipitation of the product as an off-white solid (89 mg, 79%), which was isolated by filtration. (Found: C, 44.8; H, 3.6; S, 4.8. C_24_H_25_Cl_2_PPtS requires C, 44.9; H, 3.9; S, 5.0%); ν_max_/cm^−1^ 1665, 1437, 295; ^1^H NMR (300 MHz, CD_2_Cl_2_) δ_H_ 8.0–7.5 (15H, m), 6.70 (1H, dd, ^3^*J*_Pt-H_ 67, ^2^*J*_P-H_ 10) and 1.20 (9H, s); ^31^P NMR (121 MHz, CD_2_Cl_2_) δ_P_ +29.4 (d, ^1^*J*_P-Pt_ 3524); MS (ESI^−^) *m*/*z* 641 (M–H).

#### 3.4.2. (Z)-1-Diphenylphosphino-3,3-dimethyl-2-phenylthiobut-1-ene Palladium Dichloride Complex **32**

A solution of [PdCl_2_(cod)] (38 mg, 0.13 mmol) in CH_2_Cl_2_ (5 mL) was stirred while a solution of (*Z*)-1-diphenylphosphino-3,3-dimethyl-2-phenylthiobut-1-ene **12** (50 mg, 0.13 mmol) in CH_2_Cl_2_ (5 mL) was added dropwise over 30 min. After 2 h, the mixture was reduced to 1 mL by evaporation, and the addition of diethyl ether (15 mL) led to precipitation of the product as a yellow solid (63 mg, 79%), which was isolated by filtration. (Found: C, 49.7; H, 4.4. C_24_H_25_Cl_2_PPdS•0.5 CH_2_Cl_2_ requires C, 49.4; H, 4.4%); ν_max_/cm^−1^ 1575, 1436, 289; ^1^H NMR (300 MHz, CD_2_Cl_2_) δ_H_ 8.0–7.5 (15H, m), 6.70 (1H, d, ^2^*J*_P-H_ 8) and 1.20 (9H, s); ^31^P NMR (121 MHz, CD_2_Cl_2_) δ_P_ +50.4; MS (ESI^+^) *m*/*z* 553 (M).

#### 3.4.3. (*Z*)-1-Diphenylphosphino-3,3-dimethyl-2-phenylthiobut-1-ene η^3^-allyl Palladium Chloride Complex **33**

A solution of [Pd(η^3^-allyl)Cl] (32 mg, 0.22 mmol) in CH_2_Cl_2_ (5 mL) was stirred while a solution of (*Z*)-1-diphenylphosphino-3,3-dimethyl-2-phenylthiobut-1-ene **12** (66 mg, 0.18 mmol) in CH_2_Cl_2_ (5 mL) was added dropwise over 30 min. After 2 h, the mixture was reduced to 0.5 mL by evaporation, and the addition of diethyl ether (10 mL) led to precipitation of the product as a yellow solid (54 mg, 55%), which was isolated by filtration. (Found: C, 56.7; H, 4.6. C_27_H_30_ClPPdS•0.4 CH_2_Cl_2_ requires C, 56.4; H, 5.3%); ν_max_/cm^−1^ 1599, 1435, 296; ^1^H NMR (300 MHz, CD_2_Cl_2_) δ_H_ 8.0–7.5 (15H, m), 6.70 (1H, d, ^2^*J*_P-H_ 8) and 1.20 (9H, s); ^31^P NMR (121 MHz, CD_2_Cl_2_) δ_P_ +40.9; MS (ESI^+^) *m*/*z* 523 (M–Cl).

#### 3.4.4. (*Z*)-1-diphenylphosphino-3,3-dimethyl-2-phenylthiobut-1-ene Gold Chloride Complex **34**

A solution of [Au(tht)Cl] (18 mg, 0.06 mmol) and (*Z*)-1-diphenylphosphino-3,3-dimethyl-2-phenylthiobut-1-ene **12** (21 mg, 0.06 mmol) in CH_2_Cl_2_ (2 mL) was stirred for 18 h. The mixture was reduced to 0.5 mL by evaporation, and the addition of diethyl ether (10 mL) led to precipitation of the product as a white solid (21 mg, 62%), which was isolated by filtration. (Found: C, 47.2; H, 4.1. C_24_H_25_AuClPS requires C, 47.3; H, 4.1%); ν_max_/cm^−1^ 1577, 1436, 253; ^1^H NMR (300 MHz, CD_2_Cl_2_) δ_H_ 7.5–7.0 (15H, m), 6.70 (1H, d, ^2^*J*_P-H_ 12) and 1.20 (9H, s); ^31^P NMR (121 MHz, CDCl_3_) δ_P_ +18.2; MS (ESI^+^) *m*/*z* 631 (M+Na).

#### 3.4.5. (Z)-1-Diphenylphosphino-3,3-dimethyl-2-phenylthiobut-1-ene Pentamethylcyclopentadienyl Iridium Dichloride Complex **35**

A solution of [{IrCl(μ-Cl)(η^5^-C_5_Me_5_)}_2_] (75 mg, 0.1 mmol) in CH_2_Cl_2_ (5 mL) was stirred while a solution of (*Z*)-1-diphenylphosphino-3,3-dimethyl-2-phenylthiobut-1-ene **12** (71 mg, 0.19 mmol) in CH_2_Cl_2_ (5 mL) was added dropwise over 30 min. After 2 h, the mixture was reduced to 0.5 mL by evaporation, and the addition of diethyl ether (20 mL) led to precipitation of the product as a yellow solid (84 mg, 57%), which was isolated by filtration. (Found: C, 48.1; H, 4.65. C_34_H_40_Cl_2_IrPS•1.25 CH_2_Cl_2_ requires C, 48.1; H, 4.9%); ν_max_/cm**^−^**^1^ 1648, 1437, 290; ^1^H NMR (300 MHz, CD_2_Cl_2_) δ_H_ 8.0–7.5 (15H, m), 6.70 (1H, d, ^2^*J*_P-H_ 8), 1.20 (9H, s) and 1.00 (15H, s); ^31^P NMR (121 MHz, CD_2_Cl_2_) δ_P_ −8.7; MS (ESI^+^) *m*/*z* 739 (M).

#### 3.4.6. (Z)-1-Diphenylphosphino-3,3-dimethyl-2-phenylthiobut-1-ene p-Cymene Ruthenium Dichloride Complex **36**

A solution of [{RuCl(μ-Cl)(η^6^-*p*-MeC_6_H_4_^i^Pr)}_2_] (26 mg, 0.04 mmol) in CH_2_Cl_2_ (5 mL) was stirred while a solution of (*Z*)-1-diphenylphosphino-3,3-dimethyl-2-phenylthiobut-1-ene **12** (32 mg, 0.08 mmol) in CH_2_Cl_2_ (5 mL) was added dropwise over 30 min. After 18 h, the mixture was reduced to 0.5 mL by evaporation, and the addition of diethyl ether (10 mL) led to precipitation of the product as an orange solid (39 mg, 67%), which was isolated by filtration. (Found: C, 56.9; H, 4.0. C_34_H_39_Cl_2_PRuS•0.5 CH_2_Cl_2_ requires C, 57.1; H, 5.6%); ν_max_/cm^−1^ 1637, 1436, 291; ^1^H NMR (300 MHz) δ_H_ 8.0–7.5 (19H, m), 6.70 (1H, d, ^2^*J*_P-H_ 8), 2.50 (1H, m), 1.80 (3H, s), 1.20 (9H, s) and 0.70 (6H, m); ^31^P NMR (121 MHz) δ_P_ +14.6; MS (ESI^+^) *m/z* 647 (M–Cl).

#### 3.4.7. (Z)-1-Diphenylphosphino-2-phenylthiopropene Palladium Dichloride Complex **37**

A solution of [PdCl_2_(cod)] (33 mg, 0.1 mmol) in CH_2_Cl_2_ (5 mL) was stirred while a solution of (Z)-1-diphenylphosphino-2-phenylthiopropene **13** (64 mg, 0.2 mmol) in CH_2_Cl_2_ (5 mL) was added dropwise over 30 min. After 2 h, the mixture was reduced to 0.5 mL by evaporation, and the addition of diethyl ether (10 mL) led to precipitation of the product as a yellow solid (43 mg, 73%), which was isolated by filtration. (Found: C, 49.5; H, 3.2. C_21_H_19_ClPPdS requires C, 49.3; H, 3.7%); ν_max_/cm^−1^ 1576, 1435, 296; ^1^H NMR (300 MHz, CD_2_Cl_2_) δ_H_ 8.0–7.5 (15H, m), 6.30 (1H, d, ^2^J_P-H_ 8) and 2.00 (3H, s); ^31^P NMR (121 MHz, CD_2_Cl_2_) δ_P_ +52.4; MS (ESI^+^) m/z 532 (M+Na).

### 3.5. X-ray Structure Determination of Complexes

Data were collected on a Bruker SMART diffractometer using graphite monochromated Mo Kα radiation *λ* = 0.71075 Å. The data were deposited at the Cambridge Crystallographic Data Centre and can be obtained free of charge via http://www.ccdc.cam.ac.uk/getstructures (accessed on 13 December 2023). The structure was solved by direct methods and refined by full-matrix least-squares against F^2^ (SHELXL, Version 2018/3 [35]).

#### 3.5.1. (Z)-1-Diphenylphosphino-3,3-dimethyl-2-phenylthiobut-1-ene Gold Chloride Complex **34**

Crystal data for C_24_H_25_AuClPS, *M* = 608.91, colourless prism, crystal dimensions 0.13 × 0.03 × 0.03 mm, monoclinic, space group P2_1_/c (No. 14), *a* = 18.290(6), *b* = 7.007(2), *c* = 17.660(6) Å, *β* = 96.966(6)°, *V* = 2246.7(13) Å^3^, *Z* = 4, *D*_c_ = 1.800 g cm^−3^, *T* = 125 K, *R*1 = 0.0622, *Rw*2 = 0.1417 for 2869 reflections with *I* > 2σ(*I*) and 253 variables. CCDC 2298238.

#### 3.5.2. (Z)-1-Diphenylphosphino-3,3-dimethyl-2-phenylthiobut-1-ene Pentamethylcyclopentadienyl Iridium Dichloride Complex **35**

Crystal data for C_34_H_40_Cl_2_IrPS, *M* = 774.85, yellow prism, crystal dimensions 0.30 × 0.20 × 0.20 mm, triclinic, space group P-1 (No. 2), *a* = 10.2041(15), *b* = 10.2862(15), *c* = 16.724(3) Å, *α* = 80.903(2), *β* = 82.655(2), *γ* = 65.666(2)°, *V* = 1575.5(4) Å^3^, *Z* = 2, *D*_c_ = 1.633 g cm^–3^, *T* = 125 K, *R*1 = 0.0263, *Rw*2 = 0.0717 for 4283 reflections with *I* > 2σ(*I*) and 354 variables. CCDC 2298239.

#### 3.5.3. (Z)-1-Diphenylphosphino-2-phenylthiopropene Palladium Dichloride Complex **37**

Crystal data for C_21_H_19_Cl_2_PPdS, *M* = 511.69, orange prism, crystal dimensions 0.30 × 0.15 × 0.10 mm, triclinic, space group P-1 (No. 2), *a* = 8.677(3), *b* = 11.063(4), *c* = 11.665(4) Å, *α* = 76.460(6), *β* = 87.468(6), *γ* = 71.174(5)°,*V* = 1029.8(6) Å^3^, *Z* = 2, *D*_c_ = 1.650 g cm^–3^, *T* = 125 K, *R*1 = 0.0519, *Rw*2 = 0.1335 for 2660 reflections with *I* > 2σ(*I*) and 235 variables. CCDC 2298237.

## 4. Conclusions

While thiocarbonyl ylides with other groups on the ylidic carbon undergo thermal extrusion of Ph_3_PS upon FVP at 650 °C, two examples, **10** and **11**, with hydrogen on the ylidic carbon instead undergo a novel isomerisation under the same conditions to afford useful (Z)-configured 1-diphenylphosphino-2-phenylsulfenylalkenes. The *t*-butyl compound **12** is obtained in good yield as the pure (*Z*)-isomer and behaves well as a ligand, forming a range of transition metal complexes with both bidentate binding via P and S and monodentate binding via only P. The methyl compound is obtained in lower yield mainly as the (*Z*)-isomer **13** but with a significant proportion of (*E*)-**14,** which increases upon distillation. A more limited study of its coordination chemistry resulted in the isolation of a bidentate bonded palladium complex. It is clear that while seven new complexes involving the two ligands have been isolated and characterised, including in four cases by X-ray diffraction, much more work needs to be carried out to fully exploit the potential of these simple yet versatile proligands, which are now readily available thanks to this unusual thermal rearrangement.

## Figures and Tables

**Table 1:** Geometric parameters for **10** [6], **12** [6], **31** [6], **34**, **35**, **37** and, for comparison, **38** [28].

	Bond lengths (Å)				
Compound	P–CH	CH=C	=C–S	P–M	M–S
**10**	1.739(2)	1.373(3)	1.708(2)	—	—
**12**	1.818(4)	1.316(6)	1.788(4)	—	—
**31**	1.773(14)	1.34(2)	1.825(14)	2.216(6)	2.259(5)
**34**	1.795(12)	1.332(17)	1.782(13)	2.229(3)	—
**35**	1.824(5)	1.318(7)	1.787(5)	2.3076(12)	—
**37**	1.798(8)	1.322(11)	1.796(8)	2.227(2)	2.252(2)
**38**	1.770(6)	1.346(8)	1.764(5)	2.256(1)	2.307(2)
	**Angles (°)**				
**Compound**	**P–C=C**	**C=C–S**	**=C–P–M**	**P–M–S**	**M–S–C=**
**10**	124.05(16)	122.46(16)	—	—	—
**12**	125.5(3)	115.1(3)	—	—	—
**31**	121.4(11)	118.0(10)	106.6(6)	88.59(19)	105.3(5)
**34**	128.7(10)	118.9(9)	121.2(4)	—	—
**35**	131.7(4)	119.8(4)	109.27(15)	—	—
**37**	119.6(6)	119.1(6)	107.2(3)	87.07(8)	106.8(3)
**38**	118.3(2)	120.7(2)	108.4(1)	85.1(0)	106.3(1)

## Data Availability

The data presented in this study are available in Supplementary Materials here.

## Supplementary Materials

The following are available online at https://www.mdpi.com/article/10.3390/molecules29010221/s1, Figures S1–S16: ^1^H, ^13^C and ^31^P NMR spectra of compounds **10**, **11**, **12** and **13**.
