# Supplementary material for: Thermal Rearrangement of Thiocarbonyl-Stabilised Triphenylphosphonium Ylides Leading to (Z)-1-Diphenylphosphino-2-(phenylsulfenyl)alkenes and Their Coordination Chemistry
Source: Molecules. 2023 Dec 31;29(1):221. doi: 10.3390/molecules29010221 (PMC10780699; doi:10.3390/molecules29010221)

# Supplementary Material

## Contents

Figure S1: 300 MHz $^{1}H$ NMR spectrum of **10** ($CDCl_3$)

Figure S2: 121 MHz $^{31}P$ NMR spectrum of **10** ($CDCl_3$)

Figure S3: 75 MHz $^{13}C$ NMR spectrum of **10** ($CDCl_3$)

Figure S4: 300 MHz $^{1}H$ NMR spectrum of **11** ($CDCl_3$)

Figure S5: 121 MHz $^{31}P$ NMR spectrum of **11** ($CDCl_3$)

Figure S6: 75 MHz $^{13}C$ NMR spectrum of **11** ($CDCl_3$)

Figure S7: 300 MHz $^{1}H$ NMR spectrum of **12** ($CDCl_3$)

Figure S8: 121 MHz $^{31}P$ NMR spectrum of **12** ($CDCl_3$)

Figure S9: 75 MHz $^{13}C$ NMR spectrum of **12** ($CDCl_3$)

Figure S10: 300 MHz $^{1}H$ NMR spectrum of **13** (*Z/E* 6.5:1) ($CDCl_3$)

Figure S11: 121 MHz $^{31}P$ NMR spectrum of **13** (*Z/E* 6.5:1) ($CDCl_3$)

Figure S12: 75 MHz $^{13}C$ NMR spectrum of **13** (*Z/E* 6.5:1) ($CDCl_3$)

Figure S13: 300 MHz $^{1}H$ NMR spectrum of **13** (*Z/E* 1:1) ($CDCl_3$)

Figure S14: 300 MHz $^{1}H$ NMR spectrum of **13** (*Z/E* 1:1) ($CDCl_3$) Expansions

Figure S15: 121 MHz $^{31}P$ NMR spectrum of **13** (*Z/E* 1:1) ($CDCl_3$)

Figure S16: 75 MHz $^{13}C$ NMR spectrum of **13**(*Z/E* 1:1) ($CDCl_3$)

Figure S1: 300 MHz $^{1}$H NMR spectrum of **10** ($CDCl_3$)

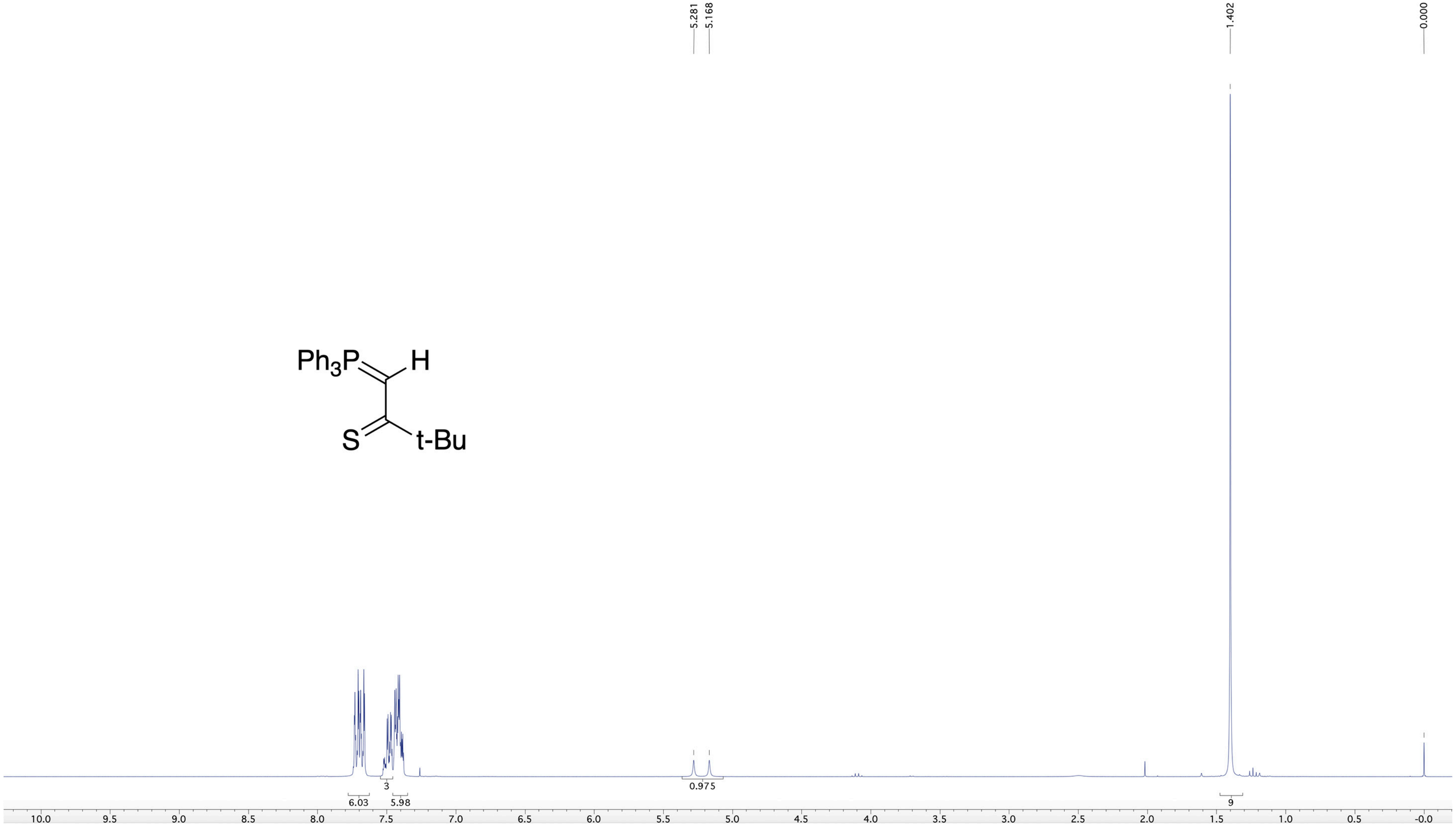

Figure S2: 121 MHz $^{31}P$ NMR spectrum of **10** ($CDCl_3$)

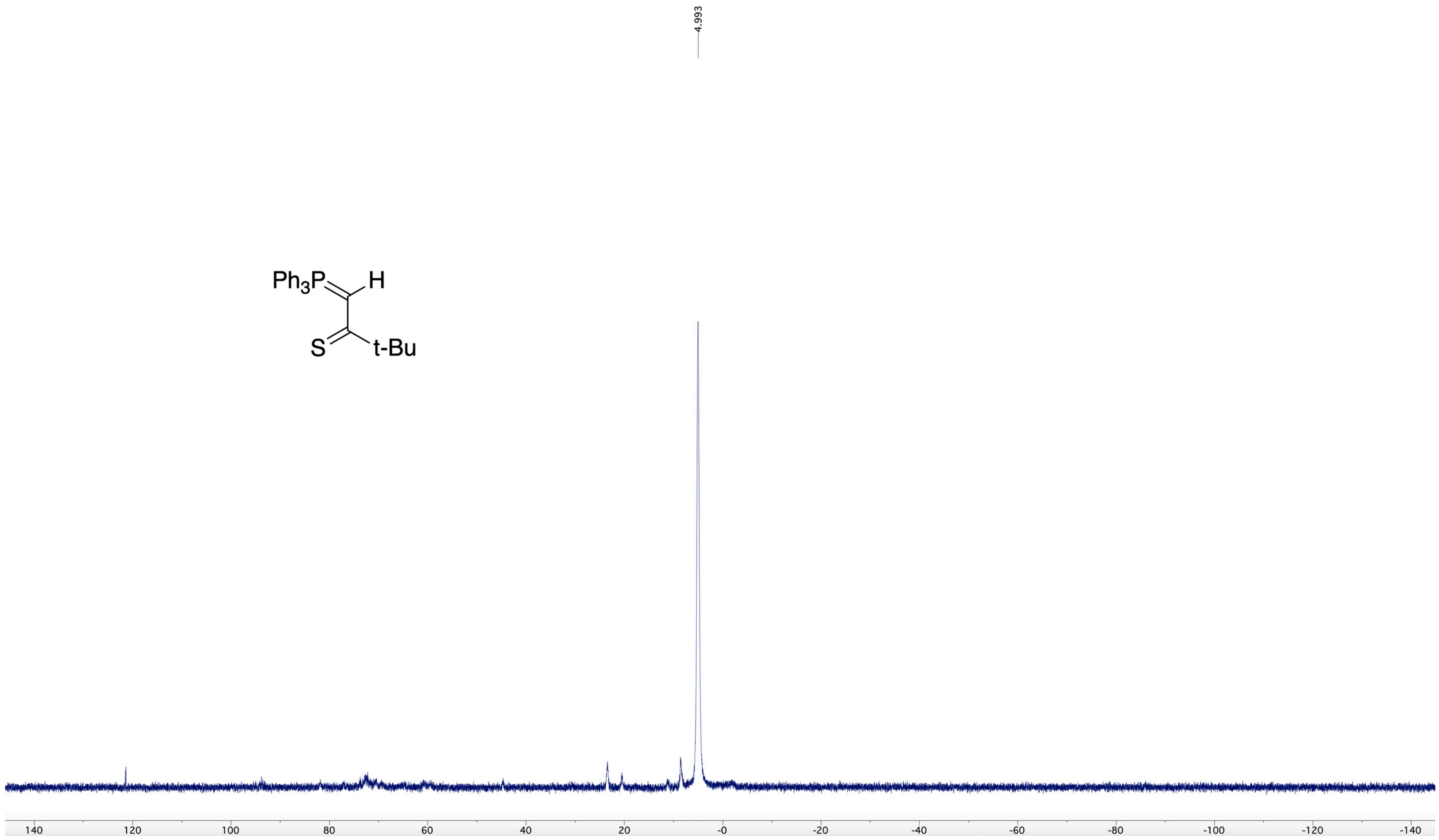

Figure S3: 75 MHz DEPTQ $^{13}C$ NMR spectrum of **10** ($CDCl_3$)

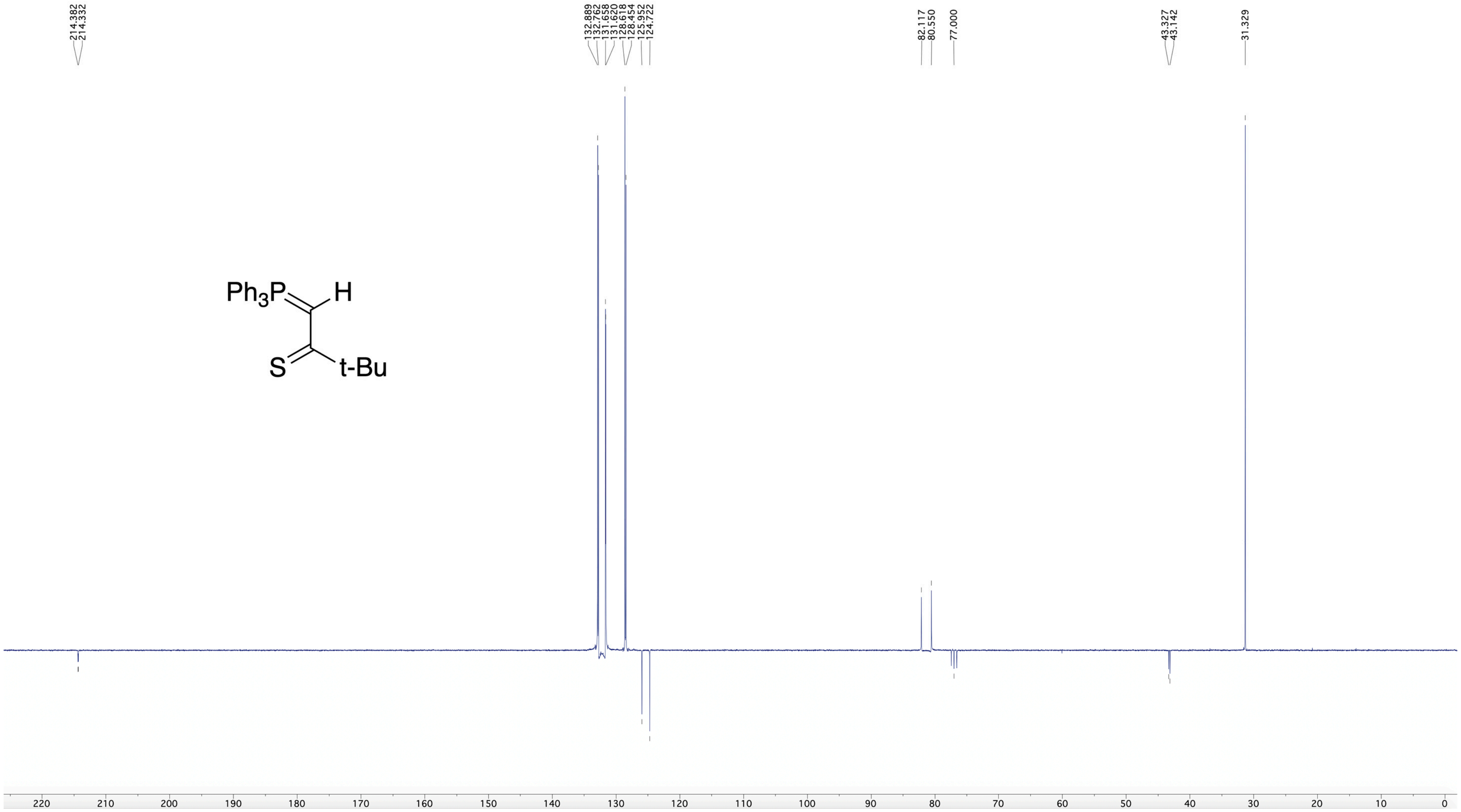

Figure S4: 300 MHz $^{1}$H NMR spectrum of **11** ($CDCl_3$)

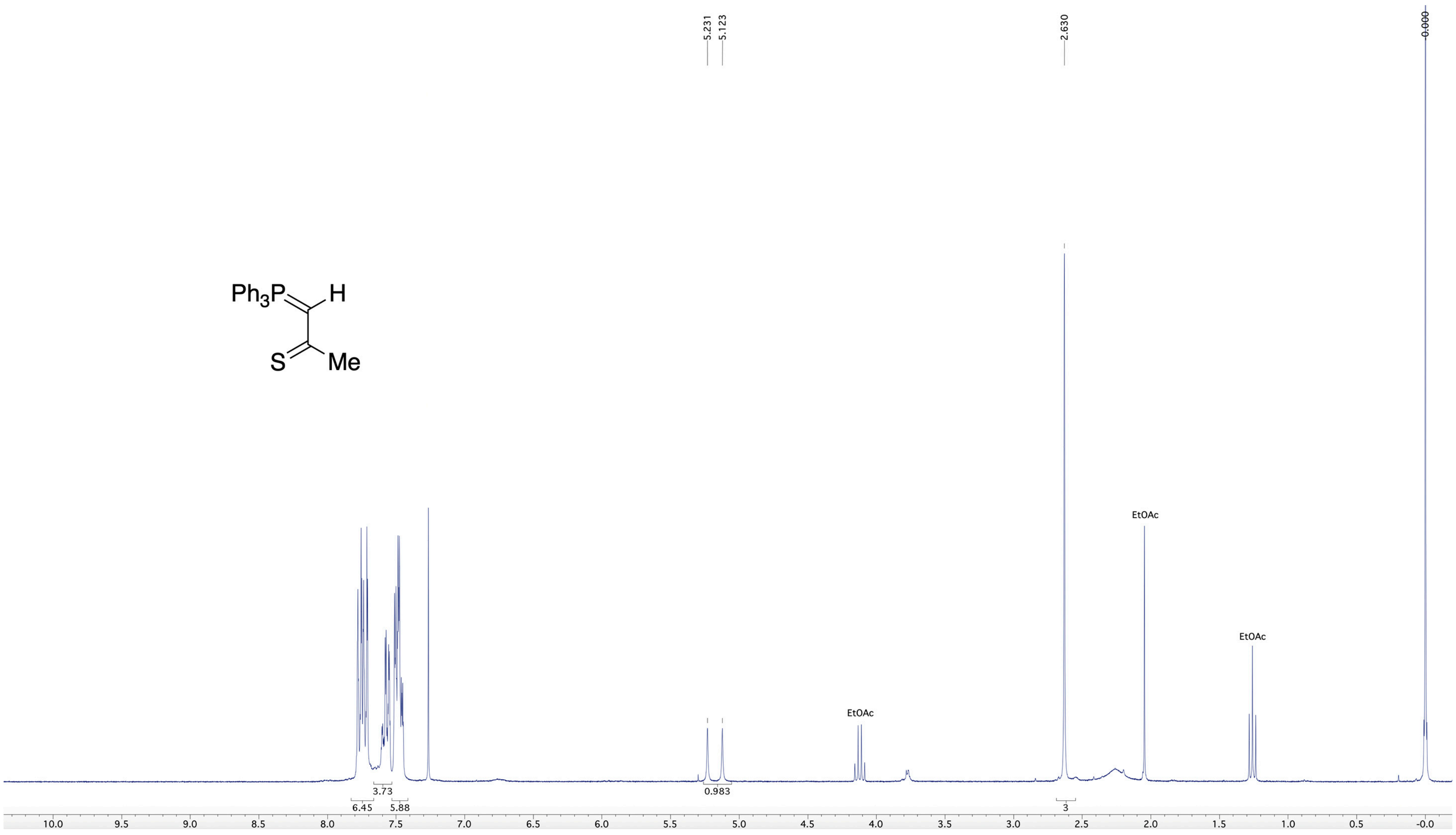

Figure S5: 121 MHz $^{31}$P NMR spectrum of **11** ($CDCl_3$)

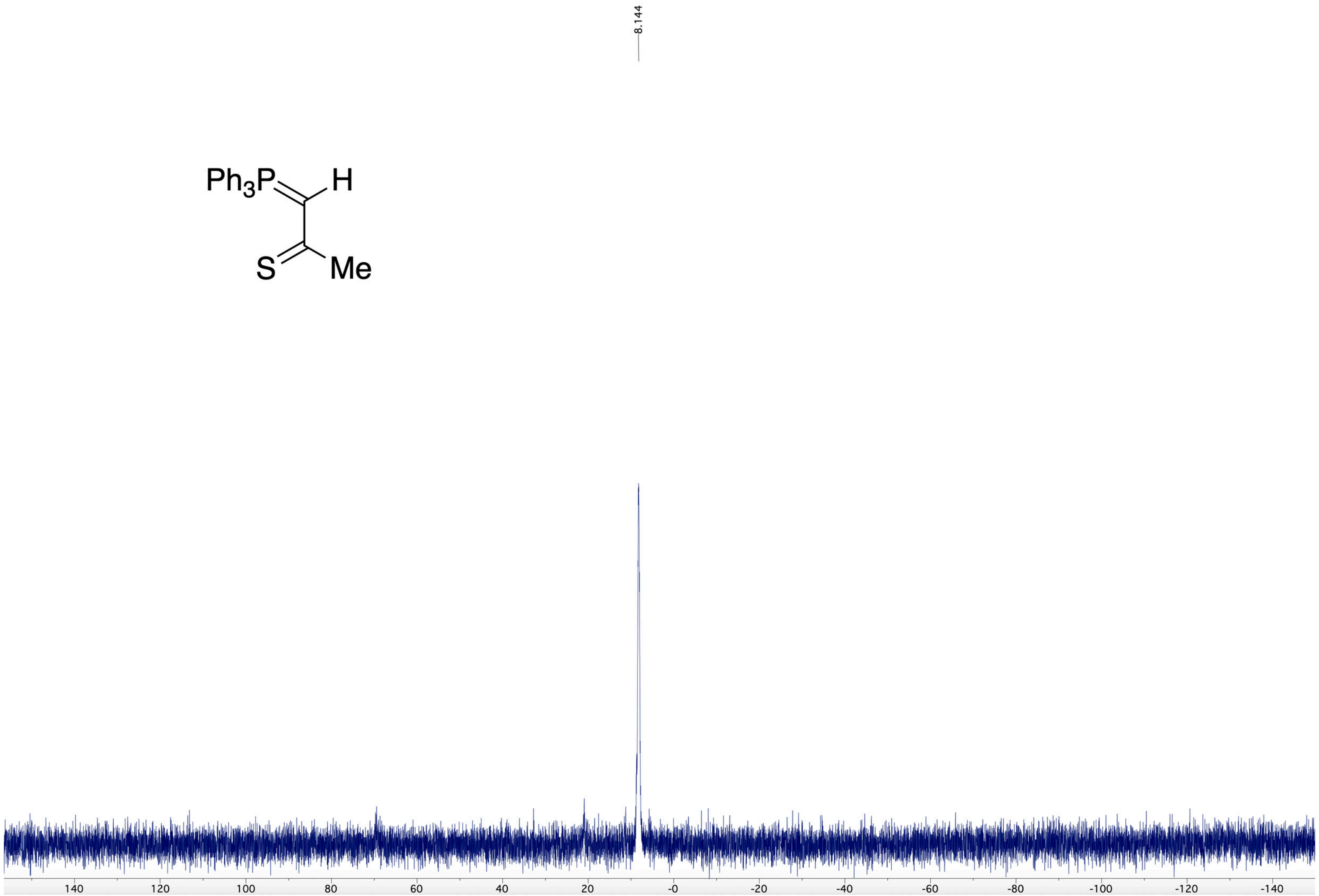

Figure S6: 75 MHz DEPTQ $^{13}C$ NMR spectrum of **11** ($CDCl_3$)

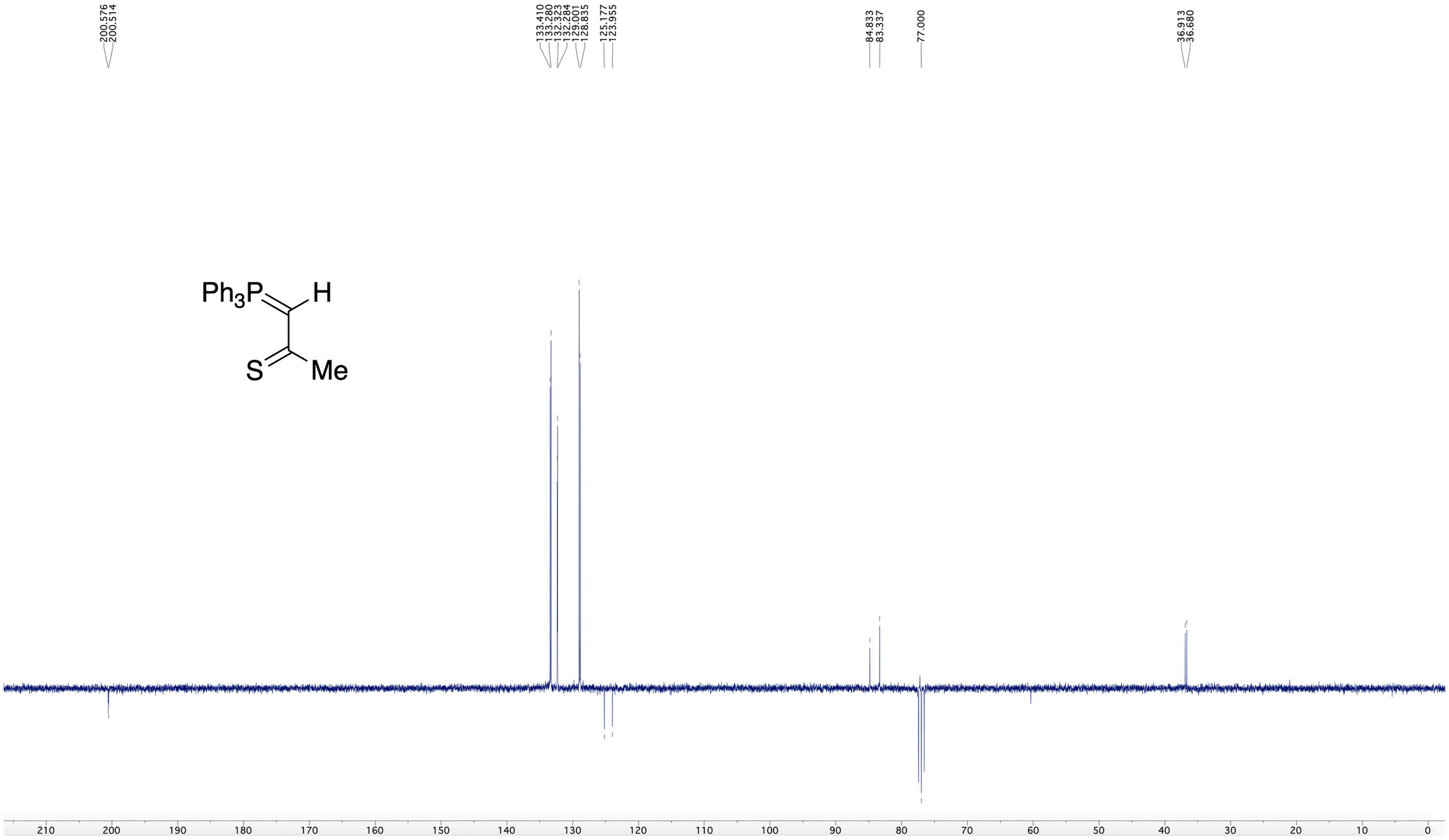

Figure S7: 300 MHz $^1$H NMR spectrum of **12** ($CDCl_3$)

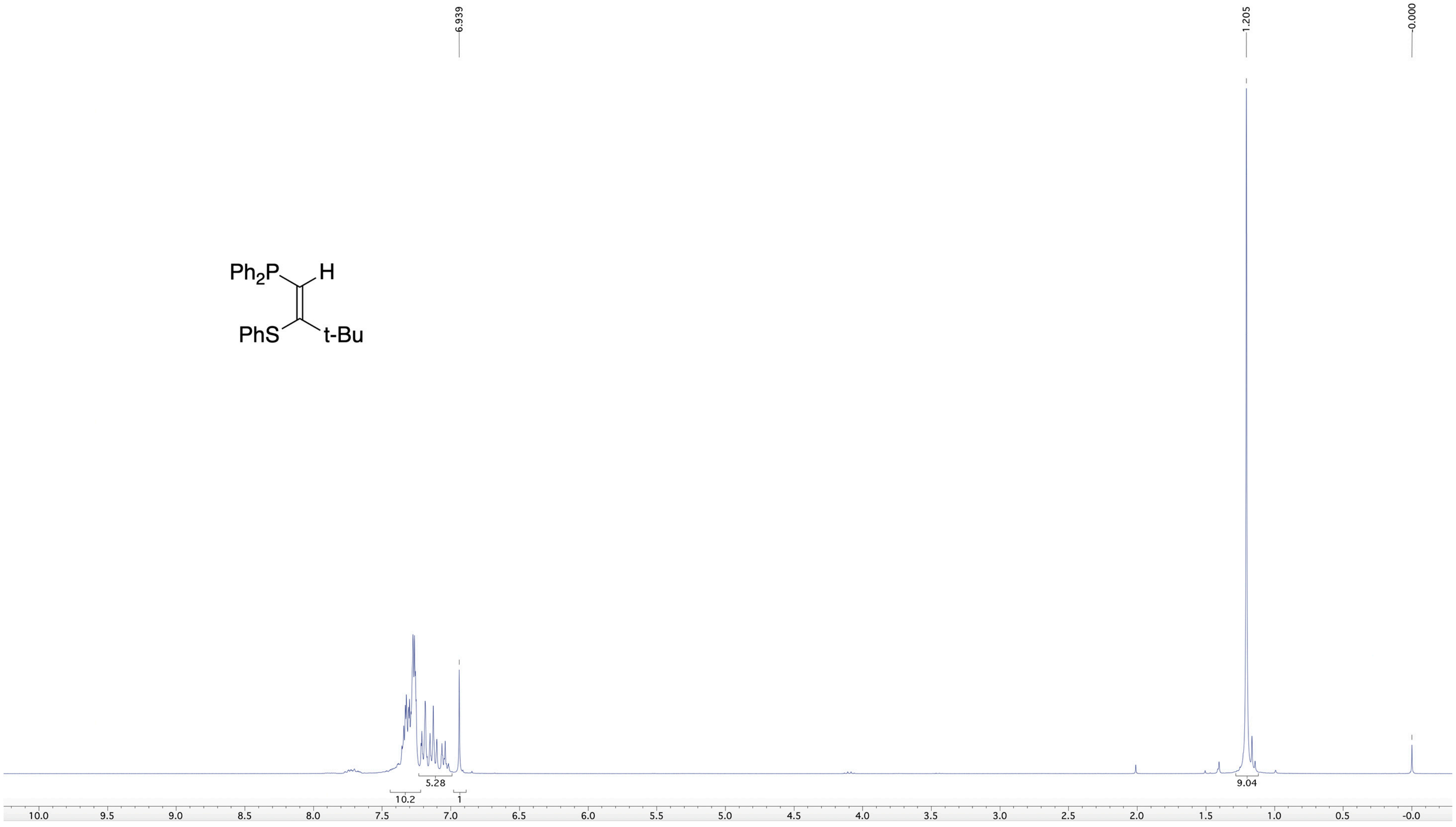

Figure S8: 121 MHz $^{31}$P NMR spectrum of **12** ($CDCl_3$)

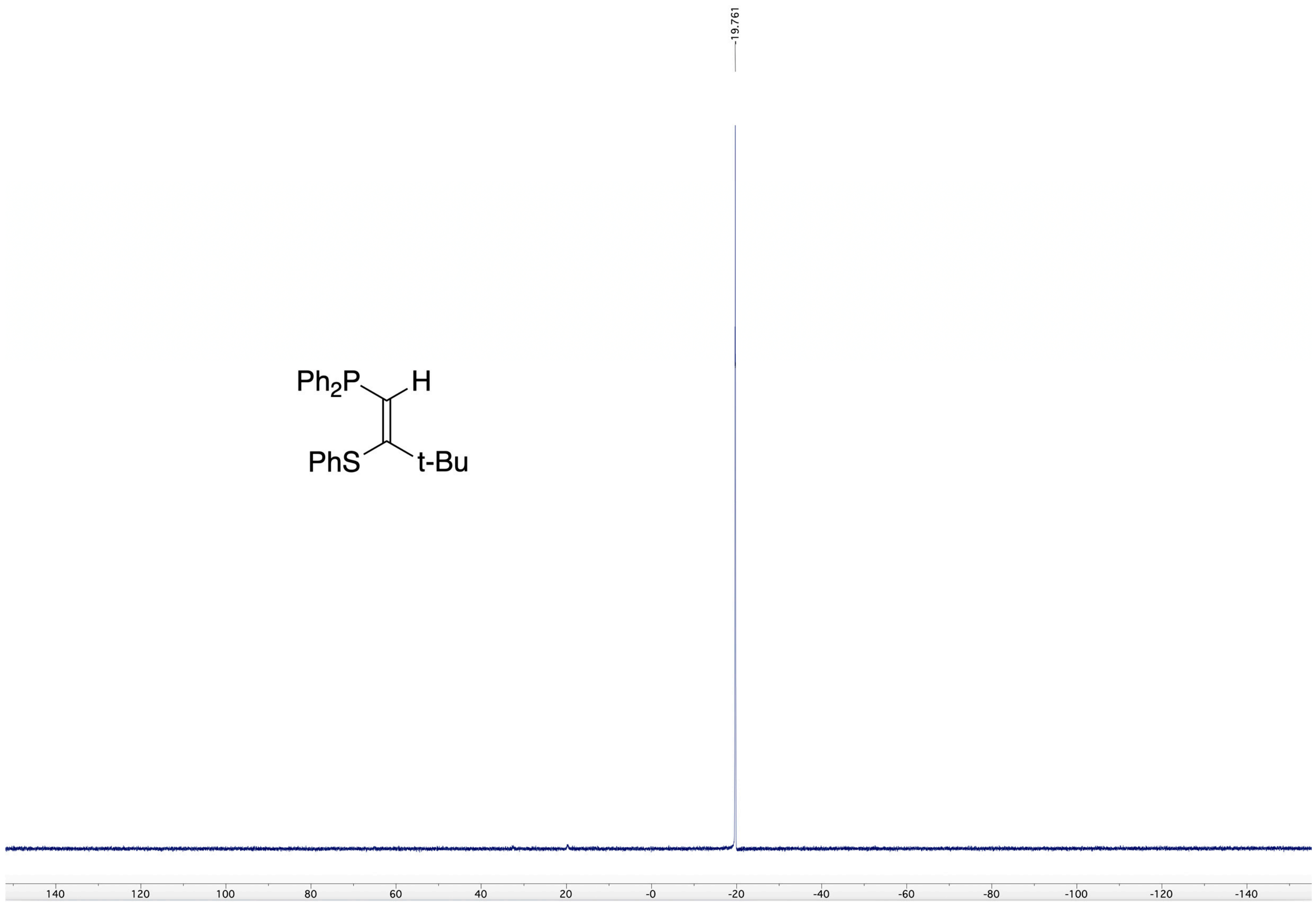

Figure S9: 75 MHz $^{13}C$ NMR spectrum of **12** ($CDCl_3$)

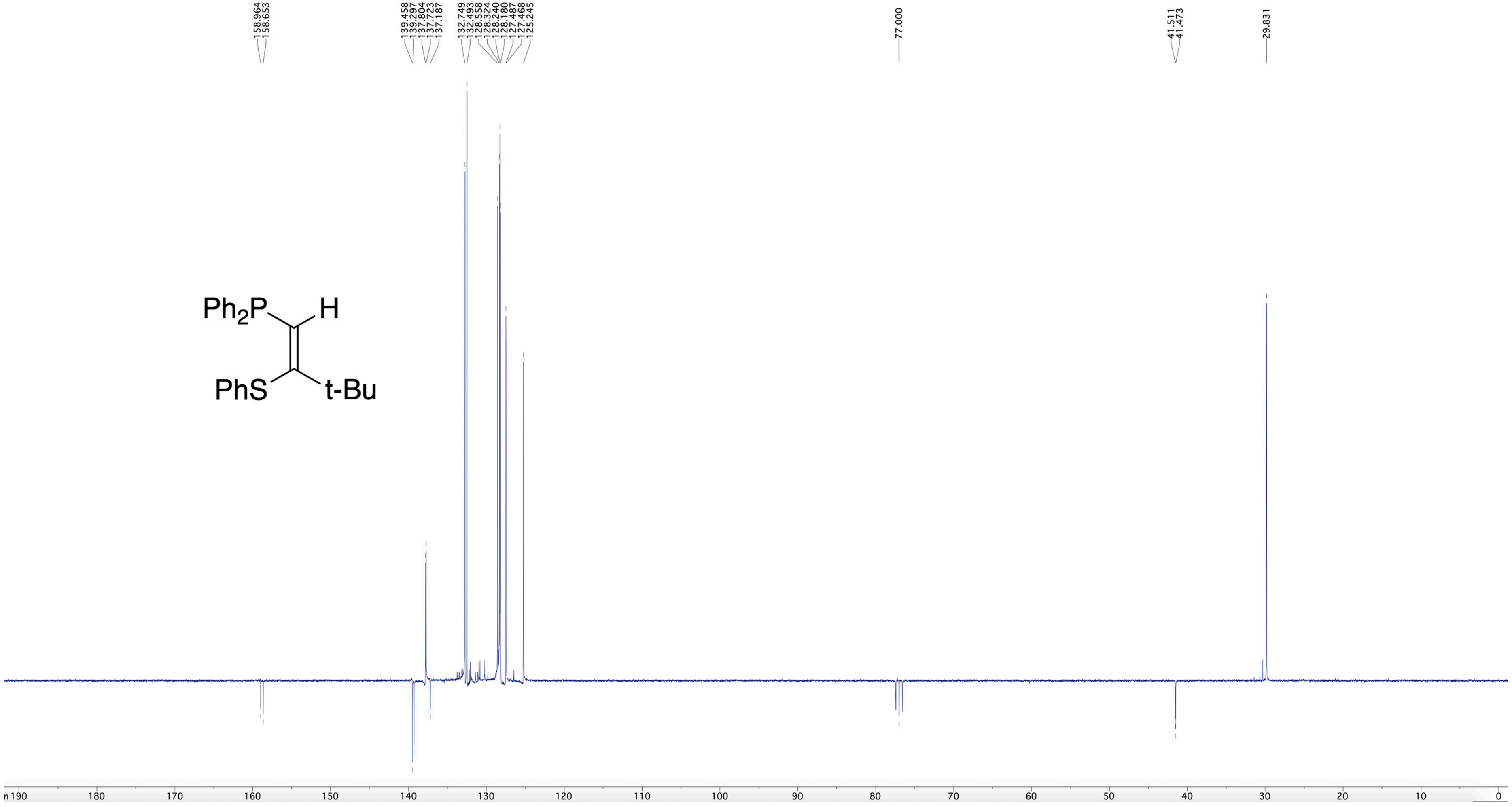

Figure S10: 300 MHz $^{1}$H NMR spectrum of **13/14** (*Z/E* 6.5:1) ($CDCl_3$)

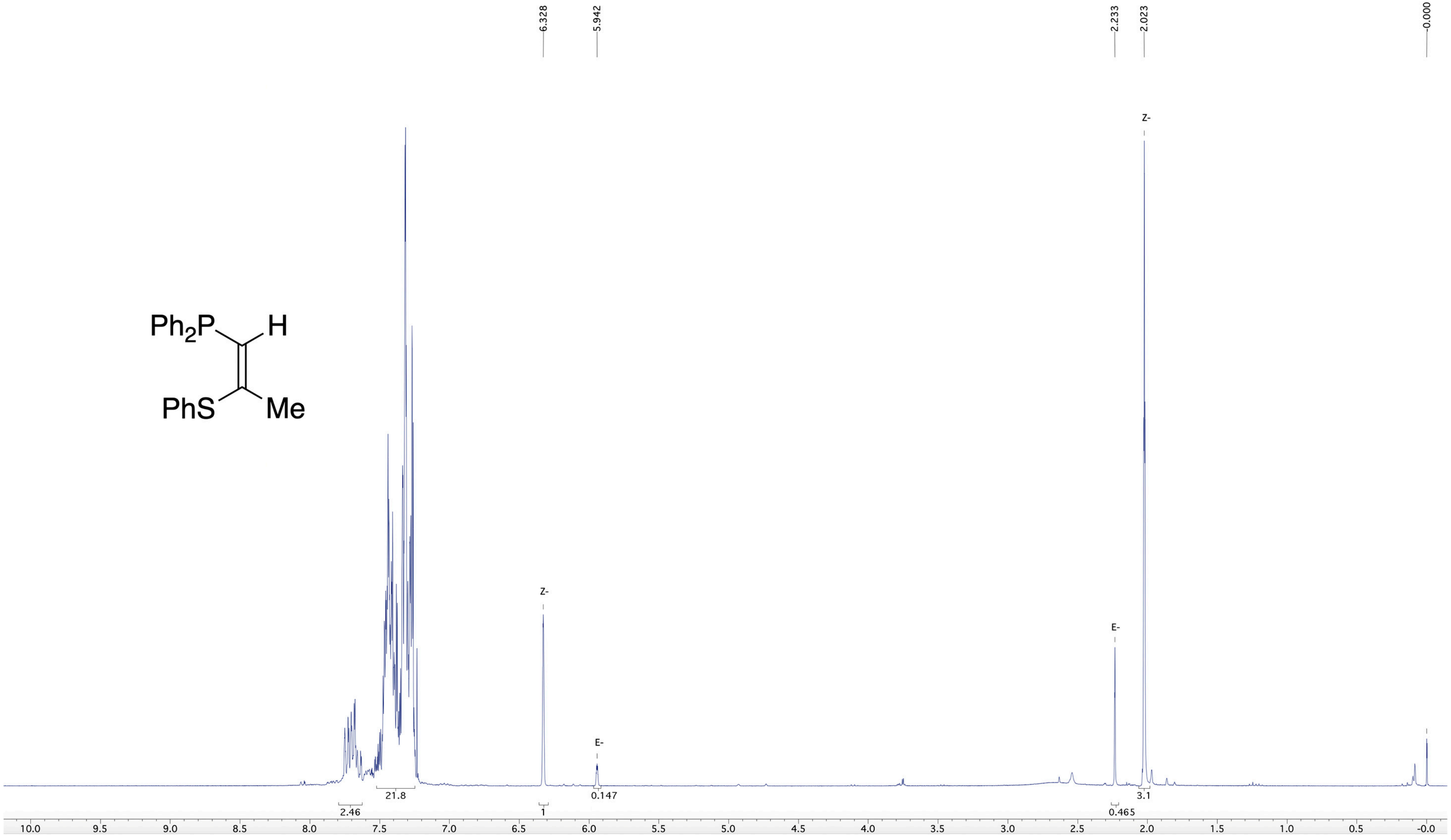

Figure S11: 121 MHz $^{31}P$ NMR spectrum of **13/14** (*Z/E* 6.5:1) ($CDCl_3$)

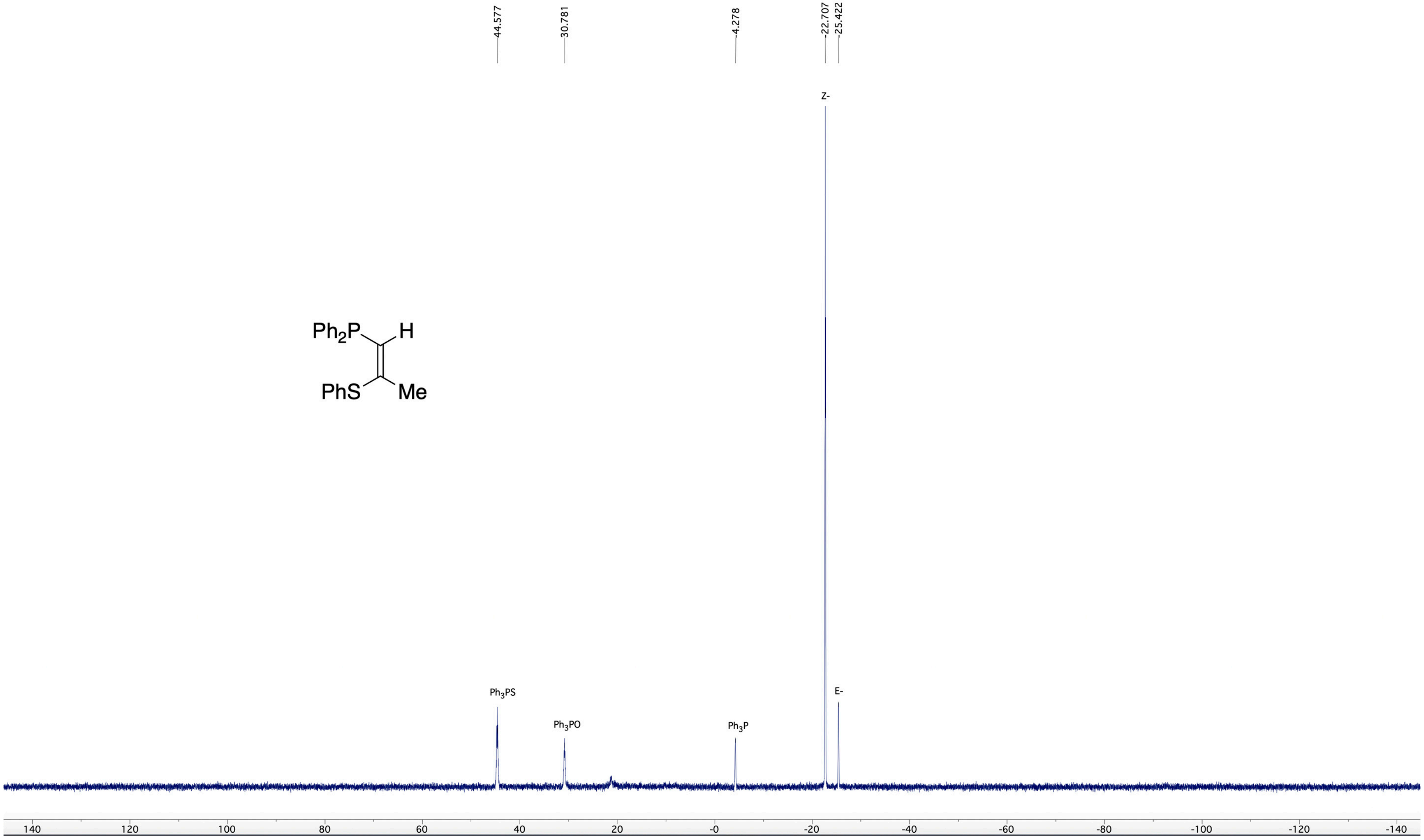

Figure S12: 75 MHz $^{13}$C NMR spectrum of **13/14** (*Z/E* 6.5:1) ($CDCl_3$)

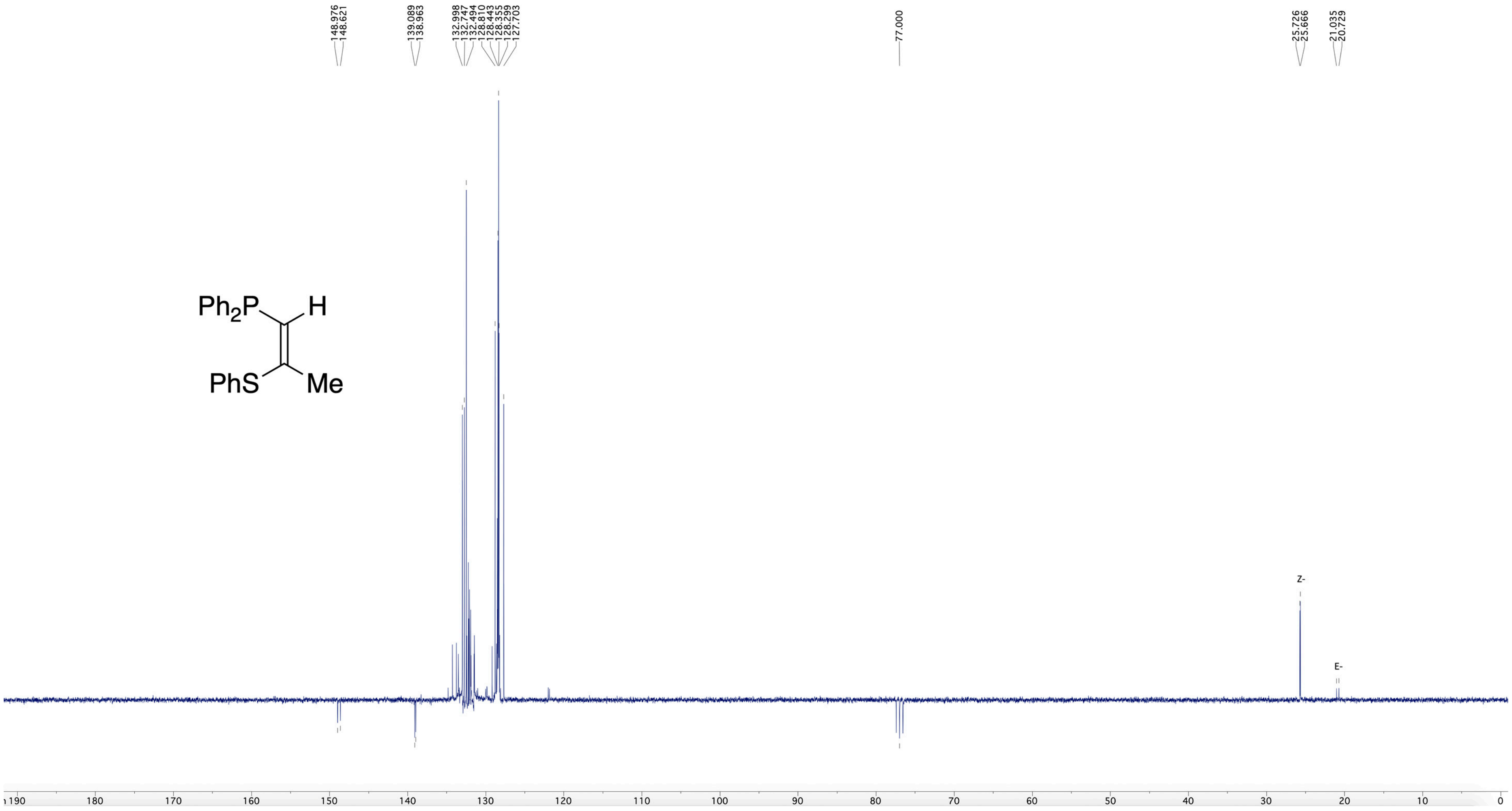

Figure S13: 300 MHz $^{1}$H NMR spectrum of **13/14** (*Z/E* 1:1) ($CDCl_3$)

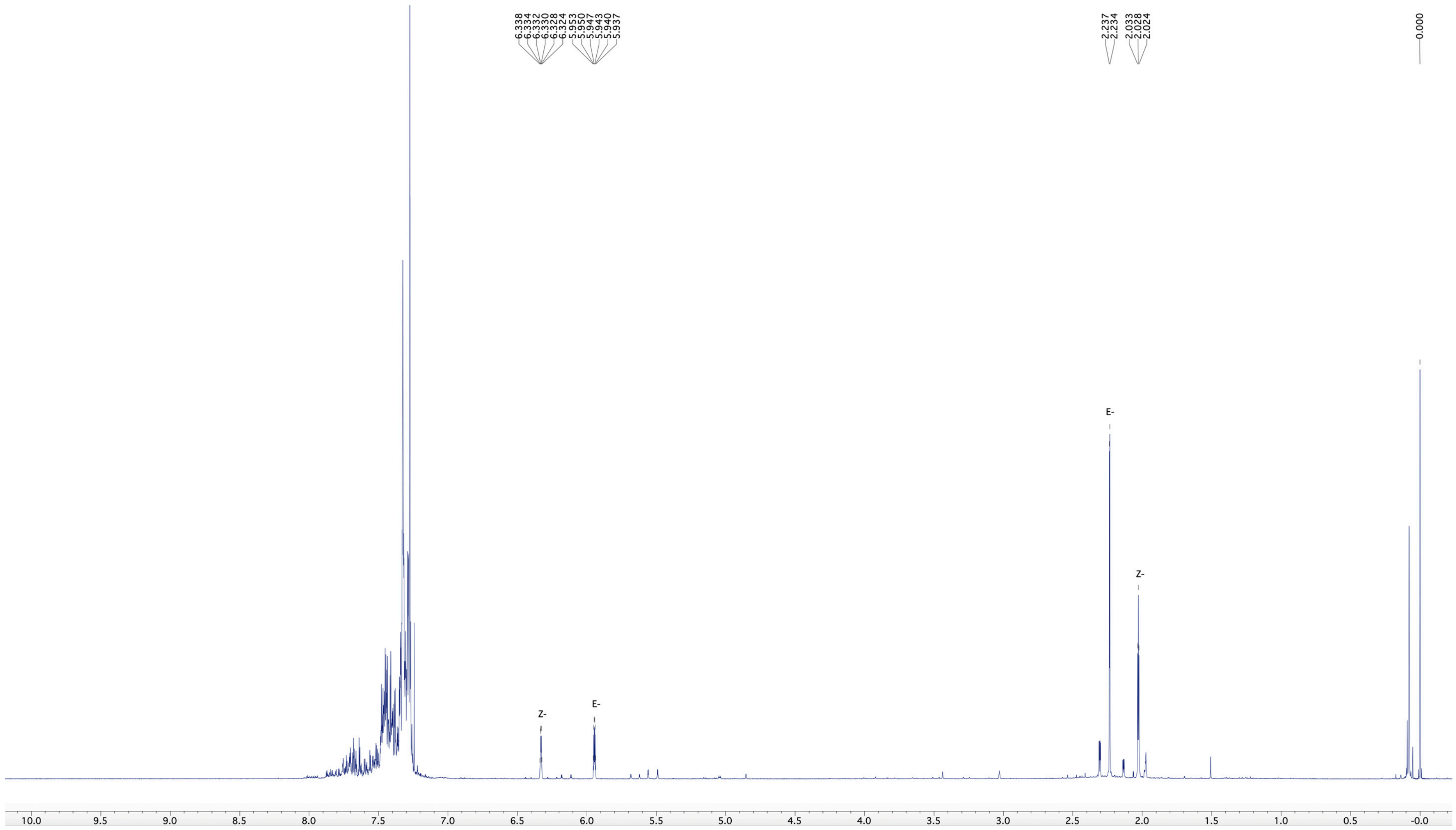

Figure S14: 300 MHz $^{1}$H NMR spectrum of **13/14** (*Z/E* 1:1) ($CDCl_3$) Expansions

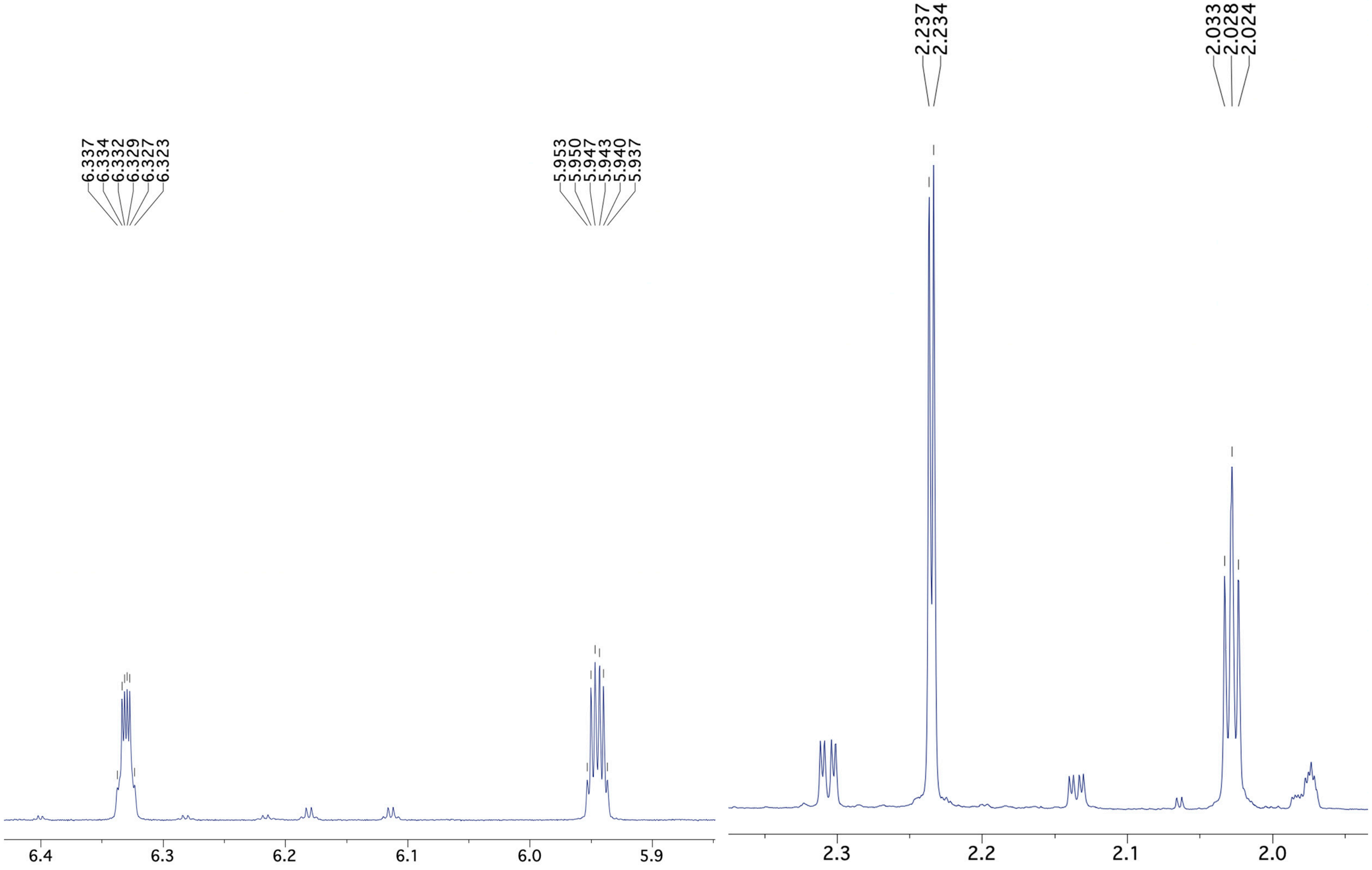

Figure S15: 121 MHz $^{31}$P NMR spectrum of **13/14** (*Z/E* 1:1) ($CDCl_3$)

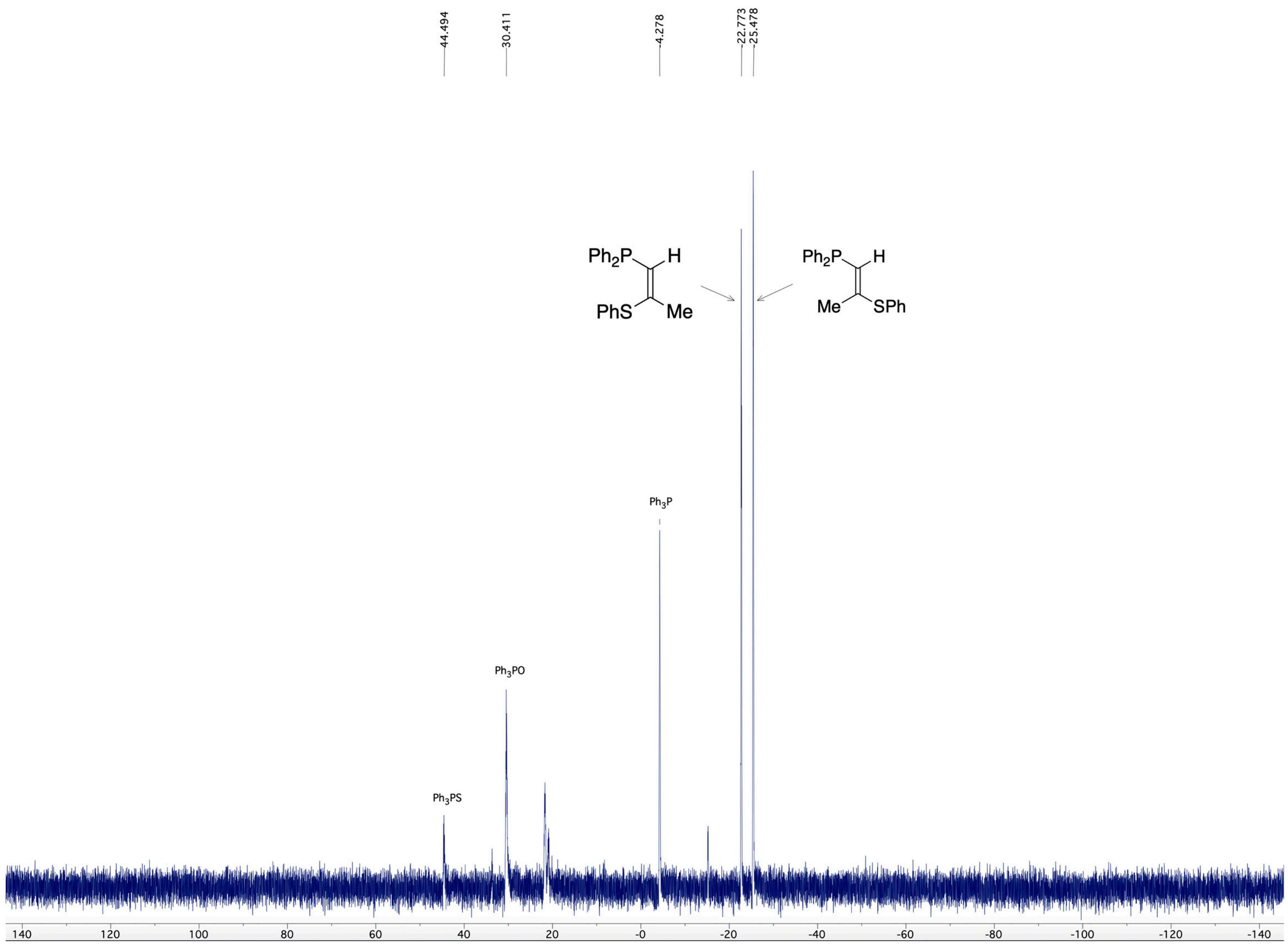

Figure S16: 75 MHz $^{13}$C NMR spectrum of **13/14** (*Z/E* 1:1) ($CDCl_3$)

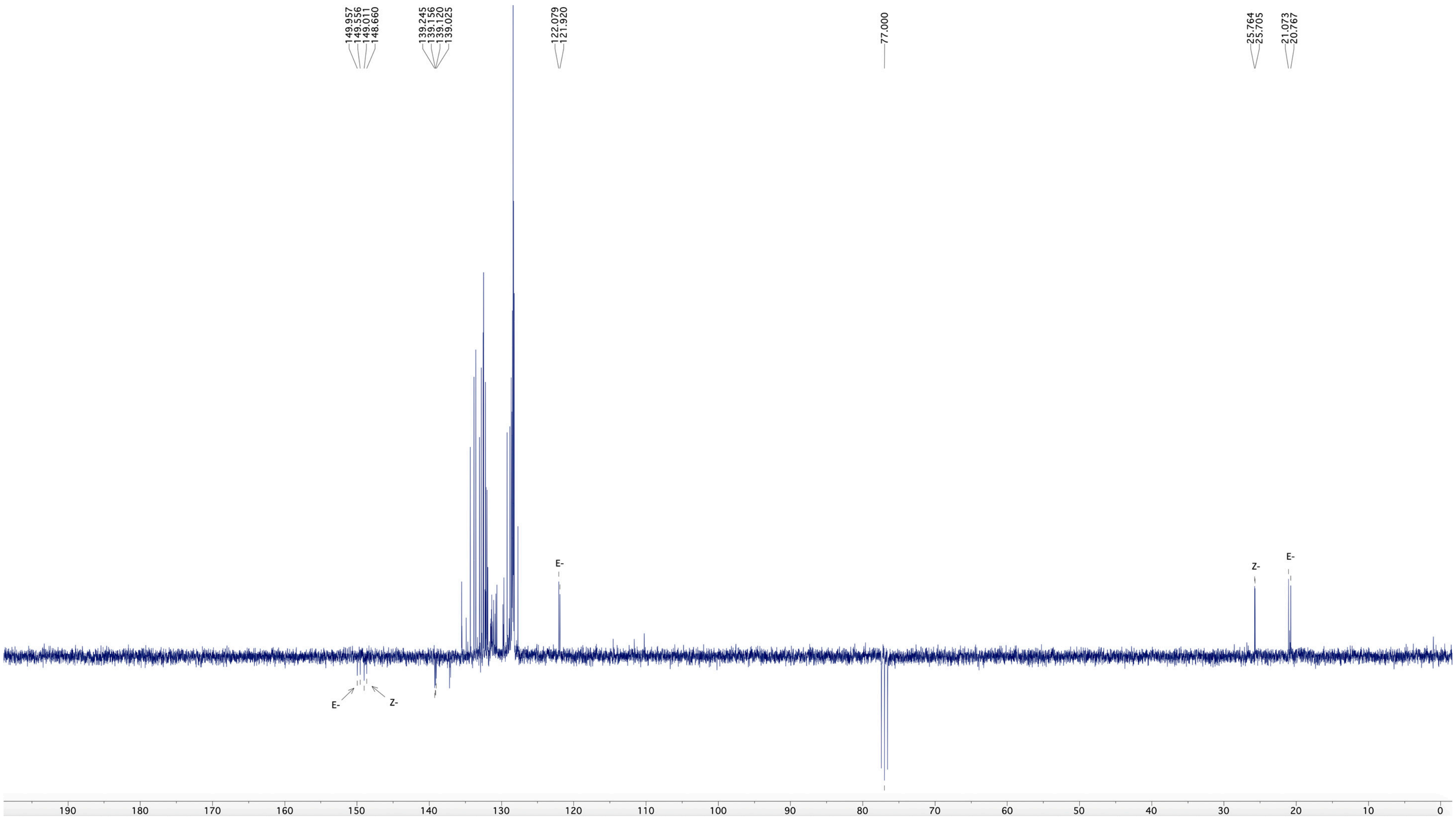

Supplement: Supplementary file 1 [file molecules-29-00221-s001.zip › 23MOL jdw SM/23MOL jdw SM.pdf]
