# Supplementary material for: Thermal Rearrangement of Thiocarbonyl-Stabilised Triphenylphosphonium Ylides Leading to (Z)-1-Diphenylphosphino-2-(phenylsulfenyl)alkenes and Their Coordination Chemistry
Source: Molecules. 2023 Dec 31;29(1):221. doi: 10.3390/molecules29010221 (PMC10780699; doi:10.3390/molecules29010221)

checkCIF/PLATON report

Structure factors have been supplied for datablock(s) 34

THIS REPORT IS FOR GUIDANCE ONLY. IF USED AS PART OF A REVIEW PROCEDURE FOR PUBLICATION, IT SHOULD NOT REPLACE THE EXPERTISE OF AN EXPERIENCED CRYSTALLOGRAPHIC REFEREE.

No syntax errors found. CIF dictionary Interpreting this report

Datablock: 34

Bond precision: C-C = 0.0179 A Wavelength=0.71075

Cell:	a=18.290(6)	b=7.007(2)	c=17.660(6)
	alpha=90	beta=96.966(6)	gamma=90
Temperature:	125 K		

	Calculated	Reported
Volume	2246.6(12)	2246.7(13)
Space group	P 21/c	P 1 21/c 1
Hall group	-P 2ybc	-P 2ybc
Moiety formula	C24 H25 Au Cl P S	C24 H25 Au Cl P S
Sum formula	C24 H25 Au Cl P S	C24 H25 Au Cl P S
Mr	608.89	608.91
Dx,g cm-3	1.800	1.800
Z	4	4
Mu (mm-1)	6.839	6.861
F000	1184.0	1184.0
F000'	1178.09	
h,k,lmax	22,8,21	21,8,21
Nref	4275	4132
Tmin,Tmax	0.781,0.814	0.163,1.000
Tmin'	0.410	

Correction method= # Reported T Limits: Tmin=0.163 Tmax=1.000
AbsCorr = MULTI-SCAN

Data completeness= 0.967 Theta(max)= 25.702

R(reflections)= 0.0622(2869) wR2(reflections)= 0.1589(4132)

S = 1.025 Npar= 253

The following ALERTS were generated. Each ALERT has the format
test-name_ALERT_alert-type_alert-level.
Click on the hyperlinks for more details of the test.

Alert level A

PLAT971_ALERT_2_A Check Calcd Resid. Dens. 0.86Ang From Au1 4.73 eA-3

Author Response: absorption effect from anistropically shaped crystal.

Alert level B

PLAT213_ALERT_2_B Atom C2 has ADP max/min Ratio ..... 4.7 oblate

Author Response: absorption effect from anistropically shaped crystal.

PLAT213_ALERT_2_B Atom C13 has ADP max/min Ratio ..... 4.5 oblate

Author Response: absorption effect from anistropically shaped crystal.

PLAT971_ALERT_2_B Check Calcd Resid. Dens. 1.00Ang From Au1 3.36 eA-3

Author Response: absorption effect from anistropically shaped crystal.

PLAT971_ALERT_2_B Check Calcd Resid. Dens. 1.03Ang From Au1 3.24 eA-3

Author Response: absorption effect from anistropically shaped crystal.

PLAT972_ALERT_2_B Check Calcd Resid. Dens. 0.94Ang From Au1 -3.20 eA-3

Author Response: absorption effect from anistropically shaped crystal.

PLAT972_ALERT_2_B Check Calcd Resid. Dens. 1.04Ang From Au1 -2.93 eA-3

Author Response: absorption effect from anistropically shaped crystal.

Alert level C

ABSTY02_ALERT_1_C An _exptl_absorpt_correction_type has been given without
a literature citation. This should be contained in the
_exptl_absorpt_process_details field.
Absorption correction given as multi-scan
PLAT213_ALERT_2_C Atom C9 has ADP max/min Ratio ..... 3.6 oblate

Author Response: absorption effect from anistropically shaped crystal.

```
PLAT213_ALERT_2_C Atom C12            has ADP max/min Ratio .....        4.0 prolat
```

Author Response: absorption effect from anistropically shaped crystal.

```
PLAT250_ALERT_2_C Large U3/U1 Ratio for Average U(i,j) Tensor ....        2.6 Note
PLAT342_ALERT_3_C Low Bond Precision on  C-C Bonds ..............    0.01791 Ang.
PLAT911_ALERT_3_C Missing FCF Refl Between Thmin & STh/L=    0.600         12 Report
PLAT977_ALERT_2_C Check Negative Difference Density on H21A      .      -0.33 eA-3
```

Alert level G

```
PLAT910_ALERT_3_G Missing # of FCF Reflection(s) Below Theta(Min).          1 Note
PLAT912_ALERT_4_G Missing # of FCF Reflections Above STh/L=  0.600        122 Note
PLAT941_ALERT_3_G Average HKL Measurement Multiplicity ..........        2.9 Low
PLAT978_ALERT_2_G Number C-C Bonds with Positive Residual Density.          0 Info
```

```
   1 ALERT level A = Most likely a serious problem - resolve or explain
   6 ALERT level B = A potentially serious problem, consider carefully
   7 ALERT level C = Check. Ensure it is not caused by an omission or oversight
   4 ALERT level G = General information/check it is not something unexpected

   1 ALERT type 1 CIF construction/syntax error, inconsistent or missing data
  12 ALERT type 2 Indicator that the structure model may be wrong or deficient
   4 ALERT type 3 Indicator that the structure quality may be low
   1 ALERT type 4 Improvement, methodology, query or suggestion
   0 ALERT type 5 Informative message, check
```

It is advisable to attempt to resolve as many as possible of the alerts in all categories. Often the minor alerts point to easily fixed oversights, errors and omissions in your CIF or refinement strategy, so attention to these fine details can be worthwhile. In order to resolve some of the more serious problems it may be necessary to carry out additional measurements or structure refinements. However, the purpose of your study may justify the reported deviations and the more serious of these should normally be commented upon in the discussion or experimental section of a paper or in the "special_details" fields of the CIF. checkCIF was carefully designed to identify outliers and unusual parameters, but every test has its limitations and alerts that are not important in a particular case may appear. Conversely, the absence of alerts does not guarantee there are no aspects of the results needing attention. It is up to the individual to critically assess their own results and, if necessary, seek expert advice.

Publication of your CIF in IUCr journals

A basic structural check has been run on your CIF. These basic checks will be run on all CIFs submitted for publication in IUCr journals (*Acta Crystallographica*, *Journal of Applied Crystallography*, *Journal of Synchrotron Radiation*); however, if you intend to submit to *Acta Crystallographica Section C* or *E* or *IUCrData*, you should make sure that full publication checks are run on the final version of your CIF prior to submission.

Publication of your CIF in other journals

Please refer to the *Notes for Authors* of the relevant journal for any special instructions relating to CIF submission.

PLATON version of 06/07/2023; check.def file version of 30/06/2023

Datablock 34 - ellipsoid plot

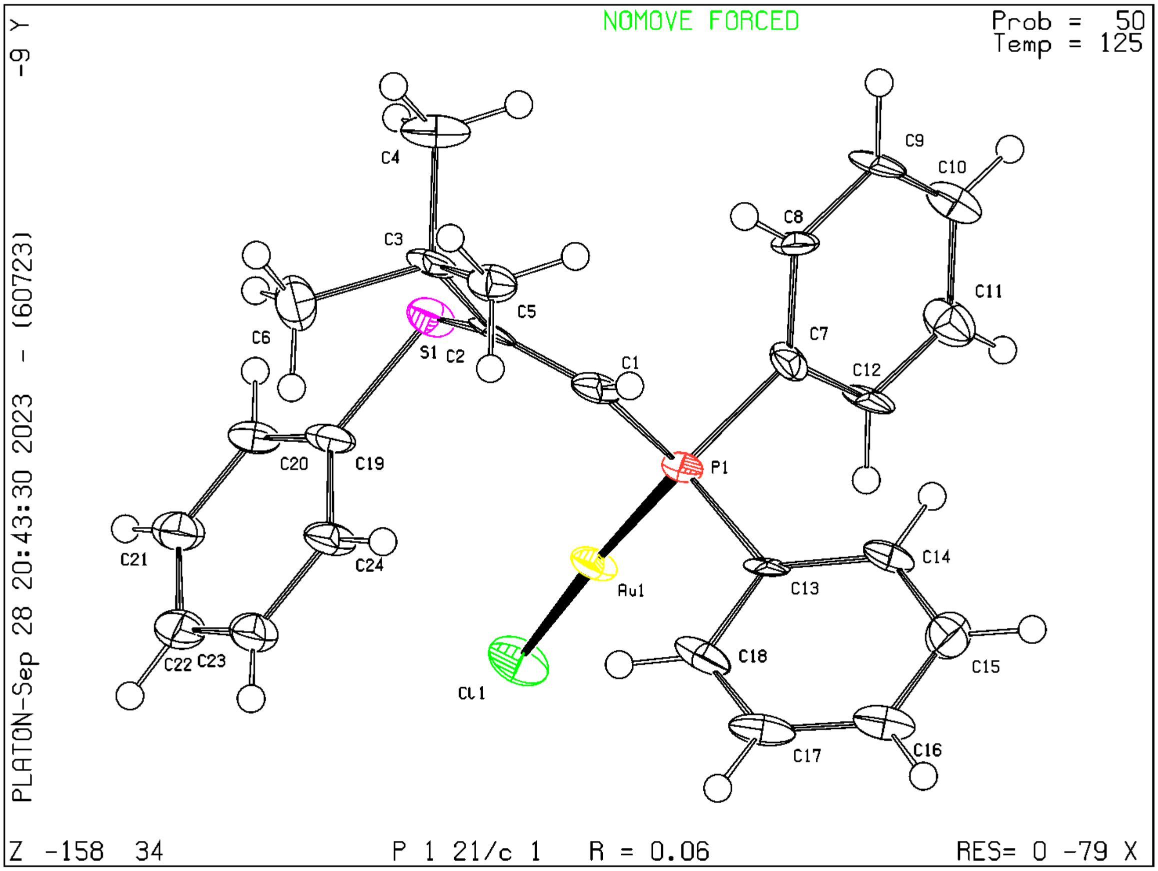

Supplement: Supplementary file 1 [file 34checkcif.pdf › 23MOL jdw SM/34checkcif.pdf]
