# Supplementary material for: Thermal Rearrangement of Thiocarbonyl-Stabilised Triphenylphosphonium Ylides Leading to (Z)-1-Diphenylphosphino-2-(phenylsulfenyl)alkenes and Their Coordination Chemistry
Source: Molecules. 2023 Dec 31;29(1):221. doi: 10.3390/molecules29010221 (PMC10780699; doi:10.3390/molecules29010221)

checkCIF/PLATON report

Structure factors have been supplied for datablock(s) 35

THIS REPORT IS FOR GUIDANCE ONLY. IF USED AS PART OF A REVIEW PROCEDURE FOR PUBLICATION, IT SHOULD NOT REPLACE THE EXPERTISE OF AN EXPERIENCED CRYSTALLOGRAPHIC REFEREE.

No syntax errors found. CIF dictionary Interpreting this report

Datablock: 35

```
Bond precision:   C-C = 0.0075 A          Wavelength=0.71075

Cell:            a=10.2041(15)      b=10.2862(15)     c=16.724(3)
                 alpha=80.903(2)    beta=82.655(2)    gamma=65.666(2)
Temperature:     125 K

                  Calculated                    Reported
Volume            1575.5(4)                     1575.5(4)
Space group       P -1                          P -1
Hall group        -P 1                          -P 1
Moiety formula    C34 H40 Cl2 Ir P S            C34 H40 Cl2 Ir P S
Sum formula       C34 H40 Cl2 Ir P S            C34 H40 Cl2 Ir P S
Mr                774.81                        774.85
Dx,g cm-3         1.633                         1.633
Z                 2                             2
Mu (mm-1)         4.546                         4.558
F000              772.0                         772.0
F000'             770.42
h,k,lmax          11,11,18                      11,11,18
Nref              4564                          4502
Tmin,Tmax         0.348,0.402                   0.743,1.000
Tmin'             0.245

Correction method= # Reported T Limits: Tmin=0.743 Tmax=1.000
AbsCorr = MULTI-SCAN

Data completeness= 0.986           Theta(max)= 23.285

                                                     wR2(reflections)=
R(reflections)= 0.0263( 4283)                        0.0725( 4502)

S = 1.023                    Npar= 354
```

```
The following ALERTS were generated. Each ALERT has the format
       test-name_ALERT_alert-type_alert-level.
Click on the hyperlinks for more details of the test.
```

Alert level B

```
THETM01_ALERT_3_B  The value of sine(theta_max)/wavelength is less than 0.575
            Calculated sin(theta_max)/wavelength =    0.5562
```

Author Response: Old data from 2003.

Alert level C

```
ABSTY02_ALERT_1_C  An _exptl_absorpt_correction_type has been given without
            a literature citation. This should be contained in the
            _exptl_absorpt_process_details field.
            Absorption correction given as multi-scan
PLAT911_ALERT_3_C Missing FCF Refl Between Thmin & STh/L=    0.556         61 Report
PLAT977_ALERT_2_C Check Negative Difference Density on H36A      .      -0.56 eA-3
PLAT977_ALERT_2_C Check Negative Difference Density on H36B      .      -0.46 eA-3
PLAT977_ALERT_2_C Check Negative Difference Density on H36C      .      -0.37 eA-3
PLAT977_ALERT_2_C Check Negative Difference Density on H38C      .      -0.33 eA-3
PLAT977_ALERT_2_C Check Negative Difference Density on H39B      .      -0.61 eA-3
PLAT977_ALERT_2_C Check Negative Difference Density on H39C      .      -0.45 eA-3
```

Alert level G

```
PLAT154_ALERT_1_G The s.u.'s on the Cell Angles are Equal ..(Note)      0.002 Degree
PLAT380_ALERT_4_G Incorrectly? Oriented X(sp2)-Methyl Moiety .....        C36 Check
PLAT380_ALERT_4_G Incorrectly? Oriented X(sp2)-Methyl Moiety .....        C38 Check
PLAT380_ALERT_4_G Incorrectly? Oriented X(sp2)-Methyl Moiety .....        C39 Check
PLAT909_ALERT_3_G Percentage of I>2sig(I) Data at Theta(Max) Still        92% Note
PLAT910_ALERT_3_G Missing # of FCF Reflection(s) Below Theta(Min).          1 Note
PLAT933_ALERT_2_G Number of HKL-OMIT Records in Embedded .res File          5 Note
PLAT941_ALERT_3_G Average HKL Measurement Multiplicity ...........        1.8 Low
PLAT978_ALERT_2_G Number C-C Bonds with Positive Residual Density.          1 Info
```

```
   0 ALERT level A = Most likely a serious problem - resolve or explain
   1 ALERT level B = A potentially serious problem, consider carefully
   8 ALERT level C = Check. Ensure it is not caused by an omission or oversight
   9 ALERT level G = General information/check it is not something unexpected

   2 ALERT type 1 CIF construction/syntax error, inconsistent or missing data
   8 ALERT type 2 Indicator that the structure model may be wrong or deficient
   5 ALERT type 3 Indicator that the structure quality may be low
   3 ALERT type 4 Improvement, methodology, query or suggestion
   0 ALERT type 5 Informative message, check
```

It is advisable to attempt to resolve as many as possible of the alerts in all categories. Often the minor alerts point to easily fixed oversights, errors and omissions in your CIF or refinement strategy, so attention to these fine details can be worthwhile. In order to resolve some of the more serious problems it may be necessary to carry out additional measurements or structure refinements. However, the purpose of your study may justify the reported deviations and the more serious of these should normally be commented upon in the discussion or experimental section of a paper or in the "special_details" fields of the CIF. checkCIF was carefully designed to identify outliers and unusual parameters, but every test has its limitations and alerts that are not important in a particular case may appear. Conversely, the absence of alerts does not guarantee there are no aspects of the results needing attention. It is up to the individual to critically assess their own results and, if necessary, seek expert advice.

Publication of your CIF in IUCr journals

A basic structural check has been run on your CIF. These basic checks will be run on all CIFs submitted for publication in IUCr journals (*Acta Crystallographica*, *Journal of Applied Crystallography*, *Journal of Synchrotron Radiation*); however, if you intend to submit to *Acta Crystallographica Section C* or *E* or *IUCrData*, you should make sure that full publication checks are run on the final version of your CIF prior to submission.

Publication of your CIF in other journals

Please refer to the *Notes for Authors* of the relevant journal for any special instructions relating to CIF submission.

PLATON version of 06/07/2023; check.def file version of 30/06/2023

Datablock 35 - ellipsoid plot

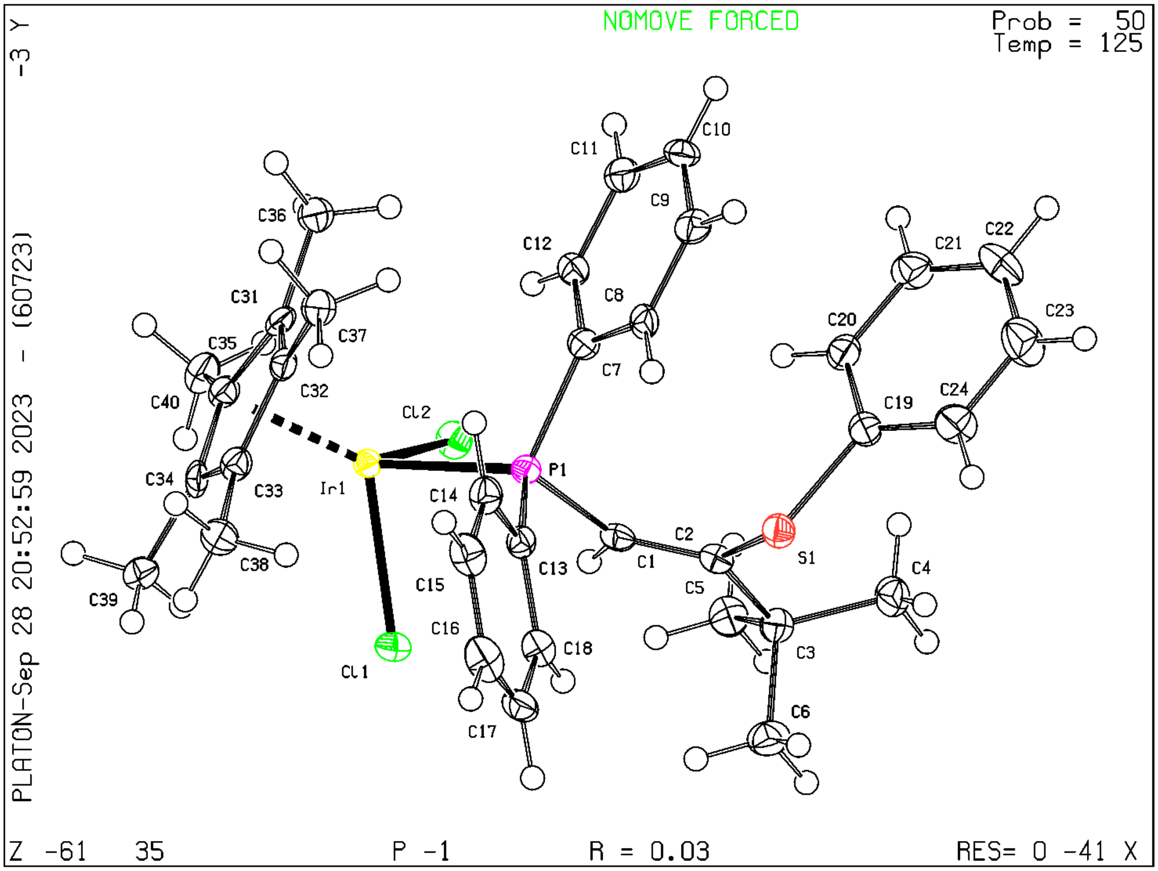

Supplement: Supplementary file 1 [file 35checkcif.pdf › 23MOL jdw SM/35checkcif.pdf]
